# Precise Synthesis of Macromolecular Architectures by Novel Iterative Methodology Combining Living Anionic Polymerization with Specially Designed Linking Chemistry

**DOI:** 10.3390/polym9100470

**Published:** 2017-09-25

**Authors:** Raita Goseki, Shotaro Ito, Yuri Matsuo, Tomoya Higashihara, Akira Hirao

**Affiliations:** 1 Polymeric and Organic Materials Department, Graduate School of Science and Engineering, Tokyo Institute of Technology, 2-12-1, Ohokayama, Meguro-ku, Tokyo 152-8552, Japan; sito@polymer.titech.ac.jp (S.I.); ymatsuo@polymer.titech.ac.jp (Y.M.); 2 Department of Chemical Science and Engineering, School of Materials Chemistry and Technology, Tokyo Institute of Technology, 2-12-1, Ohokayama, Meguro-ku, Tokyo 152-8552, Japan; 3 Department of Polymer Science and Engineering, Graduate School of Science and Engineering, Yamagata University, 4-3-16 Jonan, Yonezawa, Yamagata 992-8510, Japan; thigashihara@yz.yamagata-u.ac.jp; 4 Department of Chemical Engineering, National Taiwan University, Taipei 10617, Taiwan; 5 Department of Applied Chemistry, National Chiao Tung University, Hsinchu 30010, Taiwan

**Keywords:** living anionic polymerization, linking chemistry, μ-star polymer, high generation dendrimer-like hyperbranched polymer, exactly defined graft polymers, multiblock polymers, iterative methodology, benzyl bromide, α-phenylacrylate

## Abstract

This article reviews the development of a novel all-around iterative methodology combining living anionic polymerization with specially designed linking chemistry for macromolecular architecture syntheses. The methodology is designed in such a way that the same reaction site is always regenerated after the polymer chain is introduced in each reaction sequence, and this “polymer chain introduction and regeneration of the same reaction site” sequence is repeatable. Accordingly, the polymer chain can be successively and, in principle, limitlessly introduced to construct macromolecular architectures. With this iterative methodology, a variety of synthetically difficult macromolecular architectures, i.e., multicomponent μ-star polymers, high generation dendrimer-like hyperbranched polymers, exactly defined graft polymers, and multiblock polymers having more than three blocks, were successfully synthesized.

## 1. Introduction

Macromolecular architectures generally involve graft, star-branched, and hyperbranched polymers [1,2,3]. In this article, multiblock polymers with more than three blocks linked in a line are also added, although they are not usually categorized in macromolecular architectures. These architectural polymers have attracted increasing interest year by year because of the formation of their unique and characteristic 3D long-range morphological nanostructures and supramolecular assemblies, which can be fabricated to make nano devices important in the fields of nanoscience and nanotechnology [4,5,6,7,8,9,10,11,12,13,14]. Since the method for forming such nanostructures and supramolecular assemblies is strongly dominated by their architectures, it is essential to synthesize the well-defined macromolecular architectures.

All of the synthetic methodologies so far developed are specifically designed based on the architectures [1,2,3]. In any case, synthetic difficulty and limitation are present because multistep reactions and several reaction sites with different reactivities selectively worked in linking reaction steps are always required. For this reason, the synthesis of macromolecular architectures has long been recognized to be very difficult and still challenging even now.

To overcome the difficulty and limitation of systematically synthesizing macromolecular architectures, we have been developing a novel all-around iterative methodology combining living anionic polymerization with specially designed linking chemistry since the 1990s [15,16,17,18,19,20]. This methodology is designed in such a way that the same reaction site is always regenerated after the polymer chain introduction in each reaction sequence, and this “polymer chain introduction and regeneration of the same reaction site” sequence is repeatable. If the synthetic design operates as expected, the polymer chain can be successively, and in principle, limitlessly introduced to construct macromolecular architectures. One more important advantage of this methodology is that the use of multistep selective reactions and reaction sites with different reactivities generally required for macromolecular architecture synthesis can be completely avoided because the polymer segment is introduced one by one in each reaction step.

This iterative methodology was first applied to the synthesis of mixed arm star polymers and found to be very successful. After that, the methodology has been widely applied to the syntheses of high generation dendrimer-like hyperbranched polymers, exactly defined graft polymers, and multiblock polymers with more than three blocks. Needless to say, these polymers are synthetically very difficult even now due to their complex architectures along with structural perfection. Herein, we report on the successful synthesis of well-defined complex macromolecular architectures by the novel all-around iterative methodology combining living anionic polymerization with specially designed linking chemistry.

## 2. Macromolecular Architecture Syntheses by Iterative Methodology

### 2.1. Star Polymers ((SP)s)

The first successful macromolecular architecture synthesized by the all-around iterative methodology is a series of multicomponent (SP)s. Mixed arm (SP)s with a composition asymmetry (hereinafter, called “μ-star polymers (μ-SP)”), have recently attracted interest due to their unique morphologies on the basis of star-branched architecture [5,8,10,11,12,13,21,22,23,24,25,26,27,28,29]. However, the synthesis of well-defined (μ-SP)s with more than three different compositions is very difficult by two requirements: First, multistep selective reactions in a number that corresponds to all the different arms are required. Secondly, several reaction sites with different reactivities selectively operating for different arm introduction are required. For these requirements, the already reported methodologies using living anionic polymerization allow access only to the synthesis of two components A*_x_*B*_y_* and several ABC and ABCD (μ-SP)s [17,30,31,32,33,34,35,36,37,38,39,40,41,42,43,44,45]. Although a 5-arm ABCDE (μ-SP) was recently synthesized by the combination of living polymerization with azide-alkyne cycloaddition reaction [46], multicomponent (μ-SP) synthesis is quite limited even now.

**Scheme 1:**
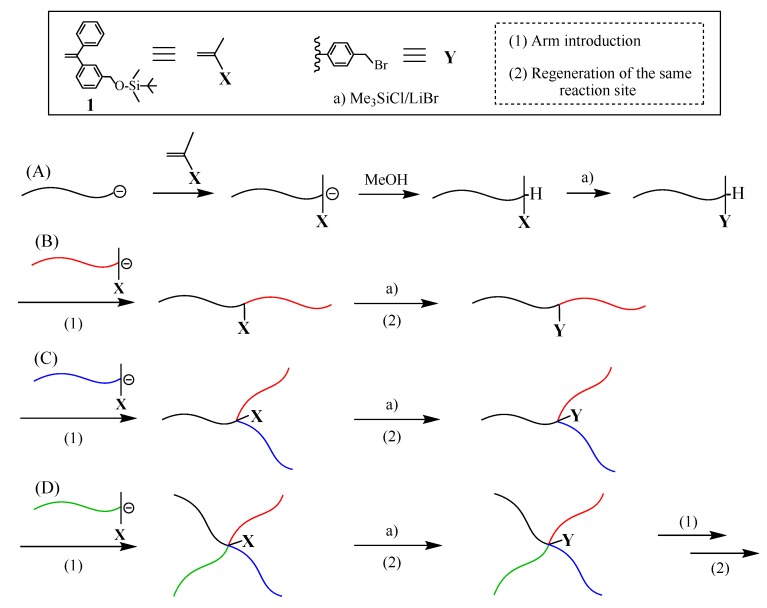
(μ-SP) synthesis by the iterative methodology. Reproduced with permission from reference [47]. Copyright 2005 American Chemical Society.

The first successful synthesis of multicomponent (μ-SP) by an iterative methodology is shown in Scheme 1 [47,48]. In the first reaction sequence, a living chain-end-**X**-functionalized polymer (A) was prepared by the end-capping of a living polymer (A) with an **X**-substituted 1,1-diphenylethylene (DPE). After the termination, the introduced **X** terminus was converted to a **Y** reaction site, which can react with a living polymer. A living chain-end-**X**-functionalized polymer (B) was prepared and in situ reacted with the resulting **Y**-functionalized polymer (A), thus forming an in-chain-**X**-functionalized diblock copolymer. After converting the **X** to **Y** reaction site, a newly prepared slightly excess amount of living chain-end-**X**-functionalized polymer (C) reacted with the **Y**-functionalized diblock copolymer to result in an ABC (μ-SP). The objective coupling product was isolated by fractional precipitation using appropriate solvent system or using preparative size exclusion chromatography (SEC). Similarly, an ABCD (μ-SP) was obtained by iterating the reaction sequence with a living chain-end-**X**-functionalized polymer (D).

The living chain-end-**X**-functionalized polymer was prepared by end-capping a living anionic polymer with 1-(3-*tert*-butyldimethylsilyloxymethylphenyl)-1-phenylethylene (**1**). The 3-*tert*-butyldimethylsilyloxymethylphenyl ω-terminus was quantitatively converted to the benzyl bromide (BnBr) reaction site with a 1:1 mixture of (CH_3_)_3_SiCl and LiBr. The results are summarized in Table 1. All of the polymers possessed the well-controlled *M*_n_ values and compositions and extremely low polydispersity indexes. The successful syntheses of the requisite ABC and ABCD (μ-SP)s are thus obvious.

As can be seen in Scheme 1, the reaction steps, (1) and (2), in each of all reaction sequences are equal to the “polymer chain (arm in this case) introduction” and the “regeneration of the same reaction site”. The living end-**X**-functionalized polymer plays as a key role in order to introduce both the arm and the **X** functionality in each sequence. Since the final (μ-SP) still has the **X** functionality convertible to the **Y** reaction site, this procedure enables further reaction sequence. Thus, the first proposed iterative methodology works very satisfactorily as designed. 

The DPE derivative, **1**, is capable of not only reacting with a living polymer but also of offering the **X** function. Thus, the use of **1** is indispensable in the methodology, but may provide a certain restriction. The living polymer, which can react with **1**, is essentially limited to highly reactive living polystyrene (PSt), polyisoprene (PIs), poly(1,3-butadiene) (PBd), or living polymers derived from their related monomers. Unfortunately, living poly(2-vinylpyridine) (P2VPy) and poly(methyl methacrylate) (PMMA) with interesting functional groups cannot be used due to their very low or no reactivities for the DPE functionality. The use of living P2VPy and (PMMA) in the linking reaction is possible to introduce P2VPy or (PMMA) segment. In such a case, however, the reaction sequence can no longer be repeated because the **X** functionality is not introduced in the linking reaction. 

**Scheme 2:**
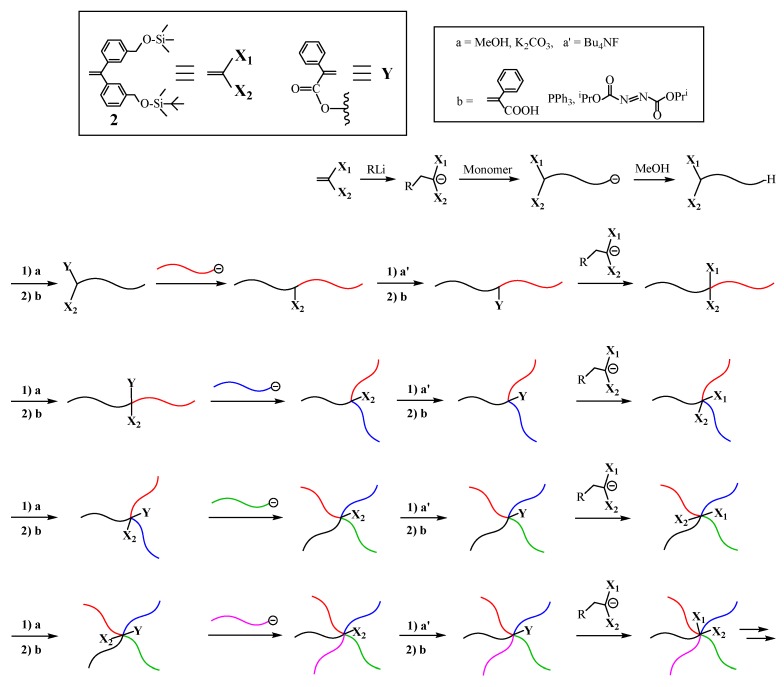
(μ-SP) synthesis by the iterative methodology using **X_1_** and **X_2_** functionalities. Reproduced with permission from reference [49]. Copyright 2012 American Chemical Society.

Very recently, a new and improved methodology has been proposed, as illustrated in Scheme 2 [49,50,51,52]. The key of this methodology is to utilize two functionalities, **X_1_** and **X_2_**, which can be deprotected in turn to separately convert to the first and second **Y** reaction sites. As the **X_1_** and **X_2_** functionalities, trimethylsilyl (TMS) and *tert*-butyldimethylsilyl (TBDMS) ethers were selected. As the starting polymer, a living polymer (A) with TMS and TBDMS termini was prepared by the living polymerization with the functional anion obtained from *sec*-BuLi and 1-(3-*tert*-butyldimethylsilyloxymethylphenyl)-1-(3-trimethylsilyloxymethylphenyl)ethylene (**2**). The first reaction sequence involves deprotection of the TMS group and the subsequent conversion of the regenerate OH group to an α-phenylacrylate (PA) reaction site, the reaction of a living polymer (B) with the reaction site, deprotection of the TBDMS group, followed by conversion to the reaction site, and the reaction of the functional anion with the reaction site to reintroduce the TMS and TBDMS ethers.

As was seen in Scheme 2, the TMS group was deprotected with MeOH containing K_2_CO_3_. The regenerated OH group was converted to the PA reaction site by the Mitsunobu reaction with α-phenylacrylic acid. The resulting (PA and TBDMS ether)-functionalized polymer was reacted with a living polymer (B) to result in an in-chain-(TBDMS ether)-functionalized AB diblock copolymer. The TBDMS group was then deprotected with (C_4_H_9_)_4_NF, followed by conversion to the PA reaction site. The functional anion, again obtained from *sec*-BuLi and **2**, reacted with the reaction site to reintroduce the TMS and TBDMS ethers.

An ABC (μ-SP) with TMS and TBDMS ethers at the core was synthesized by iterating the second sequence. The reaction sequence was further continued to yield ABCD and ABCDE (μ-SP)s. Thus, the first and second reaction sites are utilized for the arm introduction and the reintroduction of **X_1_** and **X_2_** functionalities convertible to the same reaction sites. The results are summarized in Table 2. Agreement among the calculated *M*_n_ values, compositions and those observed was satisfactory and low polydispersity indexes were attained in all the resulting (μ-SP)s. Thus, the new methodology efficiently and expectedly operates to successively synthesize ABC, ABCD, and even ABCDE (μ-SP)s. The synthesis of (μ-SP)s with more compositions would be possible, since the two silyl ethers were introduced into the 5-arm (μ-SP).

Unlike the first methodology, the advantage of this new methodology is that various living polymers, ranging from less reactive living (PMMA), P2VPy, to highly reactive living PSt, PIs, and PBd, are usable for the arm introduction because any of these living polymers readily reacts with the PA reaction site with the quantitative conversion of the reactive site. In fact, with this methodology, less reactive living (PMMA), poly(ethyl methacrylate), poly(*tert*-butyl methacrylate), poly(benzyl methacrylate), and poly(2-methoxyethyl methacrylate) all quantitatively reacted to afford a 5-arm all poly(methacrylate)-based ABCDE (μ-SP) for the first time. In the methodology, the BnBr functionality can also be used as the reaction site. However, as the conversion to the BnBr reaction site is usually carried out under strongly acidic conditions, some poly(alkyl methacrylate)s are not stable and pyridine ring in P2VPy might be formed to pyridinium salt under such conditions. 

**Scheme 3:**
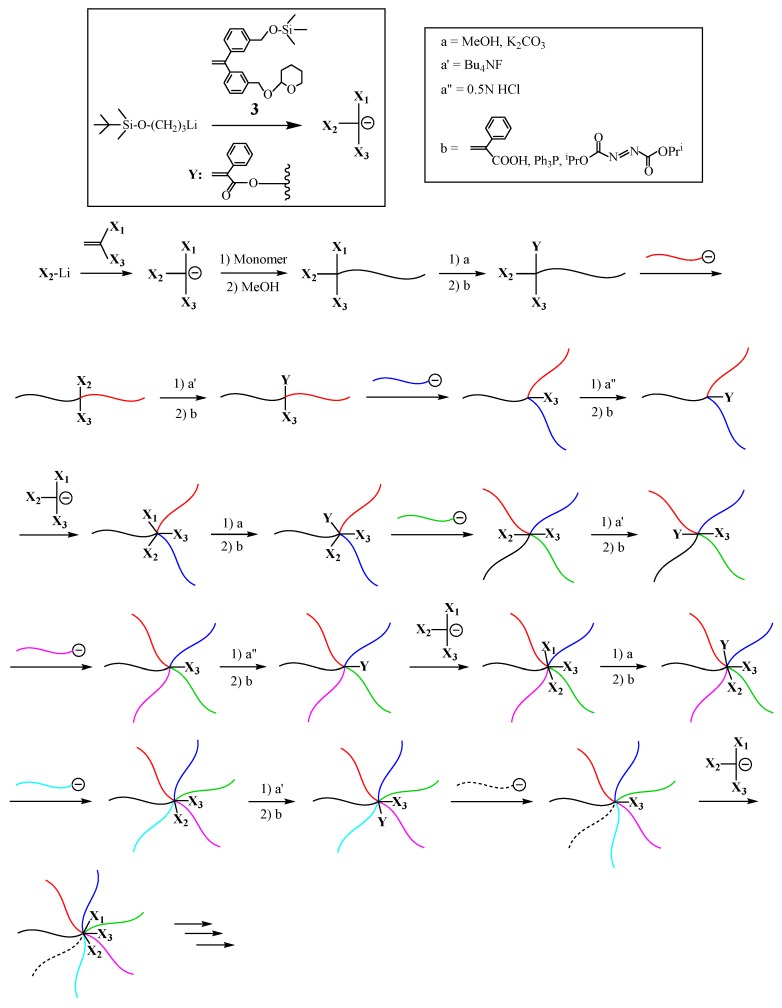
(μ-SP) synthesis by the iterative methodology using **X_1_**, **X_2_**, and **X_3_** functionalities. Reproduced with permission from reference [53]. Copyright 2013 American Chemical Society.

The new iterative methodology was further extended to the methodology with three different protected functionalities, **X_1_**, **X_2_**, and **X_3_**, as illustrated in Scheme 3 [53]. The three functionalities are designed to be sequentially converted one order to the first, second, and third **Y** reaction sites at different steps. For this design, the TMS, TBDMS, and 2-tetrahydropyranyl (THP) ethers were chosen as the **X_1_**, **X_2_**, and **X_3_** functionalities. The TMS group was first deprotected with MeOH containing K_2_CO_3_ and, on the other hand, the TBDMS and THP ethers remained unchanged. The TBDMS group was then deprotected with (C_4_H_9_)_4_NF under the conditions where the THP ether was stable. The THP ether was finally deprotected with 0.5 M HCl. The regenerated OH group in each step was converted to the PA reaction site one by one.

The starting α-terminal (TMS, TBDMS, and THP ethers)-functionalized poly(cyclohexyl methacrylate) (PCHMA) was prepared by the living polymerization of CHMA with the functional anion from 3-*tert*-butyldimethylsilyloxy-1-propyllithium and 1-(3-(2-tetrahydro-2*H*-pyranyloxy)methyl)phenyl-1-(3-trimethylsilyloxymethylphenyl)ethylene (**3**). The reaction sequence involves several steps as follows: the TMS ether deprotection and conversion to the PA reaction site, the linking with a living polymer, the TBDMS ether deprotection and conversion to the reaction site, the second linking reaction with another living polymer, the THP ether deprotection and conversion to the reaction site, and the reaction with the functional anion to reintroduce the TMS, TBDMS, and THP ethers. By iterating such the reaction sequence, the successive synthesis from ABC, ABCD, ABCDE, ABCDEF and to even ABCDEFG (μ-SP)s were successfully achieved.

As summarized in Table 3, the *M*_n_ values observed by RALLS agreed well with calculated values and low polydispersity indexes were attained in all the (μ-SP)s. Their compositions were close to calculated values. Thus, the successful syntheses of expected (μ-SP)s were evident from the results listed in this table. Since the final ABCDEFG (μ-SP) still has the THP functionality, the reaction sequence is possibly iterated. Thus, the third proposed methodology also works well as designed. The two arms are introduced by the first and second reaction sites and the three ethers are reintroduced by the third reaction site to enable further reaction sequence.

In this section, we proposed three iterative methodologies. In the first methodology, the living end-**X**-functionalized polymer was used as the building block in each reaction sequence and the next sequence was continued after converting the **X** function to the **Y** reaction site. In the last two methodologies shown in Scheme 2 and Scheme 3, the functional anions were employed to reintroduce **X_1_**, **X_2_** and **X_1_**, **X_2_**, **X_3_** functionalities, with which their reaction sequences could be iterated. It should be mentioned, however, that these methodologies are basically designed in the same concept so that each reaction sequence involves two steps of the arm introduction and regeneration of the same reaction site and can be iterated to synthesize a series of multicomponent (μ-SP)s. As often mentioned, one more important advantage of such iterative methodologies is that, in essence, the use of multistep selective reactions and several reaction sites with different reactivities generally required for the (μ-SP) synthesis are completely avoided because one arm is introduced in each reaction step.

### 2.2. Dendrimer-Like Hyperbranched Polymers ((DHBH)s)

**Figure 1:**
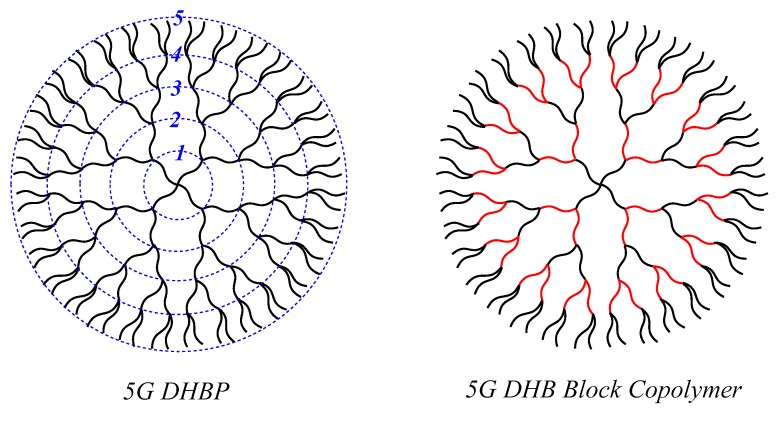
5G (DHBP) and its block copolymer.

A (DHBP) has emerged as the new class hyperbranched polymer with structural perfection since 1995 [54]. In order to clearly image the structure of (DHBP), the fifth-generation (5G) (DHBP) and its block copolymer are shown in Figure 1. These polymers are similar in branched architecture to dendrimers, but comprise several polymers connected between the layers. Accordingly, (DHBP)s are much higher in molecular weight and much larger in molecular size than dendrimers. DHPBs, believed to be globular macromolecules in shape, have many features, such as topologically specific hyperbranched architectures, hierarchic repeated layer structures, so-called “generation”, different branched densities among the core and layers, and many termini [55,56,57,58,59].

Although various (DHBP)s have been synthesized to date, most of them were limited to 2G~4G polymers with *M*_n_ values of 10^5^ g·mol^−1^ order due to several reaction steps for the introduction of additional polymer chains [60,61,62,63,64,65,66,67,68,69,70,71,72,73,74,75,76,77,78,79]. As for high-generation (≥4G) and high-molecular-weight (≥10^6^ g·mol^−1^) (DHBP)s with well-defined structures, there were reported only two examples synthesized by Gnanou et al. (7G DHB PSt with a *M*_n_ of 1.92 × 10^6^ g·mol^−1^ and 8G DHB poly(ethylene oxide) with a *M*_n_ of 6.50 × 10^5^ g·mol^−1^) and several (DHBP)s synthesized by Hirao et al. which will be described in this section [80,81,82,83,84,85,86,87].

**Scheme 4:**
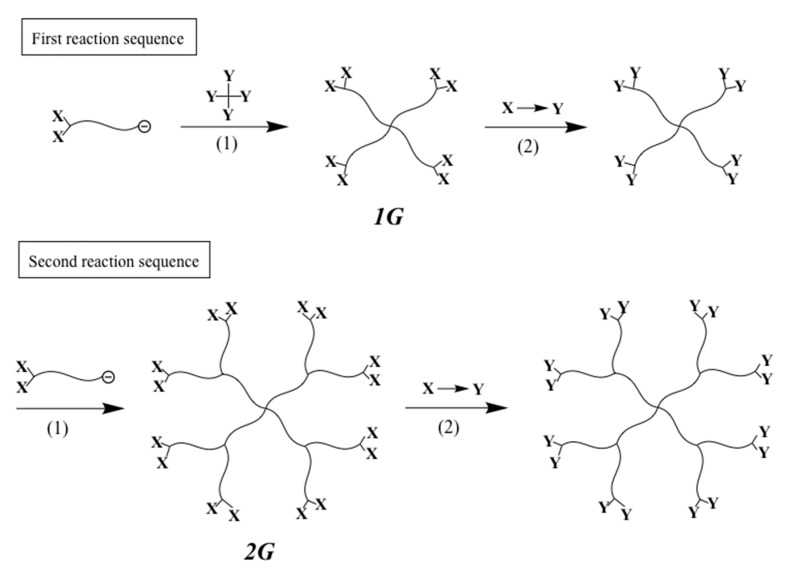
Synthesis of 1G and 2G polymers by the iterative methodology.

**Scheme 5:**
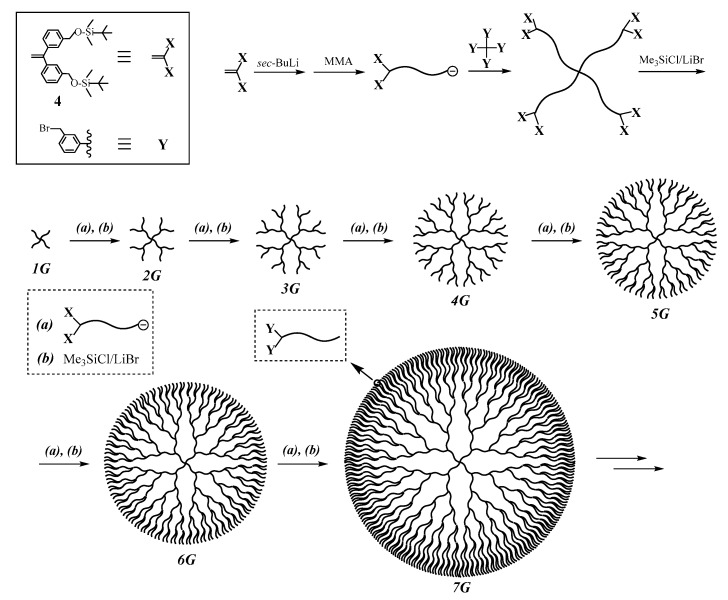
Successive synthesis of DHB (PMMA)s (1G–7G) by the iterative methodology. Reproduced with permission from reference [83]. Copyright 2005 American Chemical Society.

As can be seen in Figure 1, (DHBP)s comprise several repeating units on the basis of layer structures. It is, therefore, expected that the iterative methodology is applicable to the synthesis of (DHBP)s, as illustrated in Scheme 4 [82,83]. In the first reaction sequence, a living α-chain-end-(**X**)**_2_**-functionalized polymer reacted with a core having four **Y** reaction sites to result in a 4-arm (SP). The introduced eight **X** termini were then converted to the **Y** reaction sites. In the second sequence, the resulting (SP) with (**Y**)**_8_** termini was linked with a separately prepared living α-chain-end-(**X**)**_2_**-functionalized polymer to yield a 2G (DHBP) with sixteen **X** termini. Thus, the living α-chain-end-(**X**)**_2_**-functionalized polymer is used as the building block in each reaction sequence. As was seen, the (DHBP) was obtained from the second sequence. The linking of the living polymer with either the core compound or the polymer with (**Y**)**_8_** termini and the conversion to the **Y** reaction site are exactly equal to (1) the arm introduction and (2) regeneration of the reaction site, respectively. Since the 2G (DHBP) possesses the sixteen **Y** reaction sites, the reaction sequence can be repeated. Indeed, it was repeated to successively synthesize 3G, 4G, 5G, 6G, and even 7G (DHBP)s (see Scheme 5). As can be seen, the polymer obtained in one reaction sequence before becomes the starting polymer in the next sequence [83]. 

The building block was prepared by the living polymerization of MMA with the initiator obtained from *sec*-BuLi and 1,1-bis(3-*tert*-butyldimethylsilyloxymethylphenyl)ethylene (**4**). Similar to the previous methodologies, the introduced TBDMS ether (**X**) is readily and quantitatively converted to the BnBr (**Y**) reaction site. The linking reactions proceeded with ~100% efficiencies at −40 °C for a few hours with 2 equivalents of living α-chain-end-(TBDMS)_2_-functinalized (PMMA) to afford 2G, 3G, and 4G polymers. The use of 3–5 equivalents of living (PMMA) and longer reaction times to 48 h were needed to complete the reaction, yielding 5G, 6G, and 7G polymers.

As summarized in Table 4, the resulting DHB (PMMA)s all had well-controlled *M*_n_ values in agreement with those calculated and low polydispersity indexes, (*M*_w_/*M*_n_ ≤ 1.03). Agreement between the calculated values and those observed in end-functionality is excellent in each of all samples. The 7G DHB (PMMA) was a huge macromolecule with a *M*_w_ value of 1.97 × 10^6^ g·mol^−1^ and consisted of 508 (PMMA) segments having 512 BnBr termini, which enable further synthetic sequence of more generation (DHBP)s. Thus, the synthesis of well-defined (DHBP)s was quite successful by employing the iterative methodology using the living α-chain-end-(TBDMS ether)_2_-functionalized polymer as the building block. This success also made it possible to synthesize other (DHBP)s made up of poly(*tert*-butyl methacrylate) (P^t^BMA), PSt, and a mixture of these two polymers by a slightly modified procedure using the PA reaction site. Several amphiphilic generation-based block copolymers with PSt and poly(methacrylic acid) layers could be obtained from the DHB block copolymers consisting of (P^t^BMA) and PSt, followed by hydrolysis of the (P^t^BMA) segments [86].

**Scheme 6:**
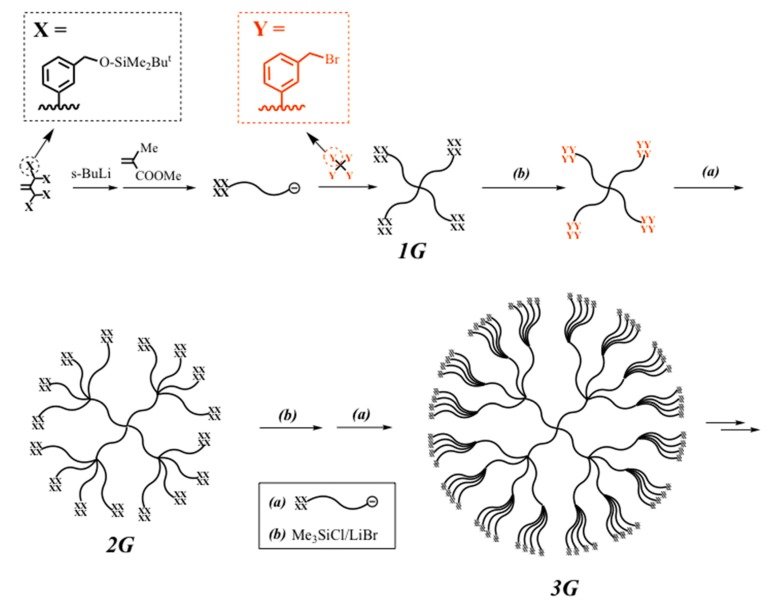
Successive synthesis of highly dense DHB (PMMA)s (1G–3G). Reproduced with permission from ref. [84]. Copyright 2006 WILLEY-VCH Veriag GmbH & Co. KGaA, Weinheim, Germany.

**Figure 2:**
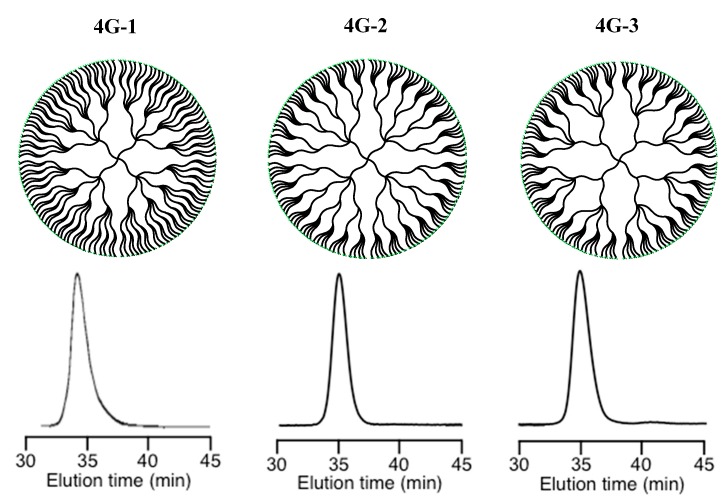
SEC Profiles of **4G-1**, **4G-2**, and **4G-3** DHB (PMMA)s. Reproduced with permission from reference [85]. Copyright 2009 American Chemical Society.

A highly dense 3G DHB (PMMA) with four polymer chains branched at the core as well as in the two layers was synthesized by the iterative methodology with a living α-chain-end-(TBDMS ether)_4_-functionalized (PMMA) (see Scheme 6) [84]. The resulting polymer had a *M*_n_ value of 9.30 × 10^5^ g·mol^−1^ and 256 BnBr termini (see Table 5). Further synthesis was not possible in this case. Instead, three different 4G (DHBP)s shown in Figure 2 could be synthesized by using the living α-chain-end-(TBDMS)_2_-functionalized (PMMA) in either the second, third, or fourth reaction sequence. All of the polymers were around 2 million in *M*_n_ value with 512 BnBr termini even in the 4G polymers (see also Table 5) [85].

We demonstrated the effectiveness of the iterative methodology for the synthesis of high-generation and high-molecular-weight (DHBP)s. The methodologies shown in Scheme 4, Scheme 5 and Scheme 6 are basically the same as that shown in Scheme 1, except for the use of different building blocks and the core compound. Since highly reactive BnBr or PA termini are present in the intermediate and final polymers, various useful functionalities, such as perfluoroalkanes, olefins (double bonds), acetylenes (triple bonds), alkoxy and hydrosilanes, epoxide, thiol, phosphine, ferrocene, and monosaccharide residues, etc., can be introduced at any generation, internal, and external positions [55]. As a result, well-defined functional hyperbranched and nano-size globular macromolecules with many potential applications are synthesized. Novel and interesting morphological nanostructures and supramolecular assemblies will also be expected from generation-based DHB block polymers [55,56,58,76,77,79]. 

### 2.3. Graft Polymers ((GP)s)

**Figure 3:**
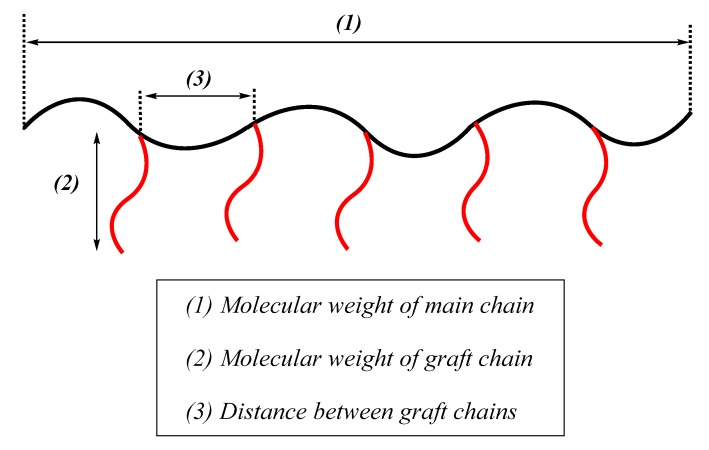
Three structural parameters to define graft polymer. Reproduced with permission from reference [88]. Copyright 2006 WILLEY-VCH Veriag GmbH & Co. KGaA, Weinheim, Germany.

As illustrated in Figure 3, a (GP) is defined by three structural parameters: (1) molecular weight of the main chain; (2) molecular weight of the graft chain; and (3) distance between the graft chains. An ideal (GP), in which the three parameters are perfectly controlled, is termed an “exactly defined graft polymer (EDGP)” [88]. Although the physical and mechanical properties as well as morphology of (GP) are significantly influenced by such three structural parameters, their relationships have not been enough elucidated due to the synthetic difficulty of (EDGP) [1,2,89,90,91]. In practice, various (GP)s have been synthesized so far, but all of them are not (EDGP)s, except for the PIs–*graft*–PSt reported by Hadjichristidis et al. [88] and several graft polymers synthesized by Hirao et al., which will be introduced in this section [92,93,94,95,96,97]. 

**Scheme 7:**
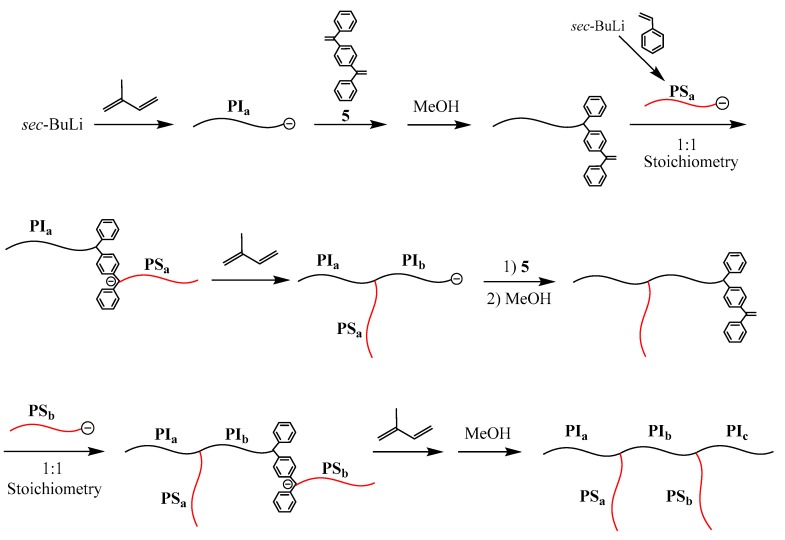
Synthesis of (EDGP) with two PSt graft chains. Reproduced with permission from reference [92]. Copyright 2009 American Chemical Society.

As shown in Scheme 7, a living PIs reacted with 1,4-bis(phenylethenyl)benzene (**5**) to introduce a DPE terminus. After the termination, a living PSt reacted with the DPE terminus to prepare a PIs–*block*–PSt in-chain anion, from which isoprene was polymerized. The resulting living 3-arm (SP) reacted with **5** to again introduce the DPE terminus. The same reaction sequence was iterated to synthesize a PIs–*graft*–PSt with two PSt graft chains. In the resulting polymer, the three structural parameters are perfectly controlled by the living polymerization of either isoprene or styrene in each step. Although the synthesis of (EDGP)s with more than three graft chains is possible by iterating the reaction sequence, it seems difficult because an exact stoichiometry is required in each reaction between living PSt and the DPE terminus. If the stoichiometry is deviated in the reaction, many side products difficult in separation are by-produced. Later, an (EDGP) having five graft chains was successfully synthesized by an improved methodology based on the termination procedure [92]. 

**Figure 4:**
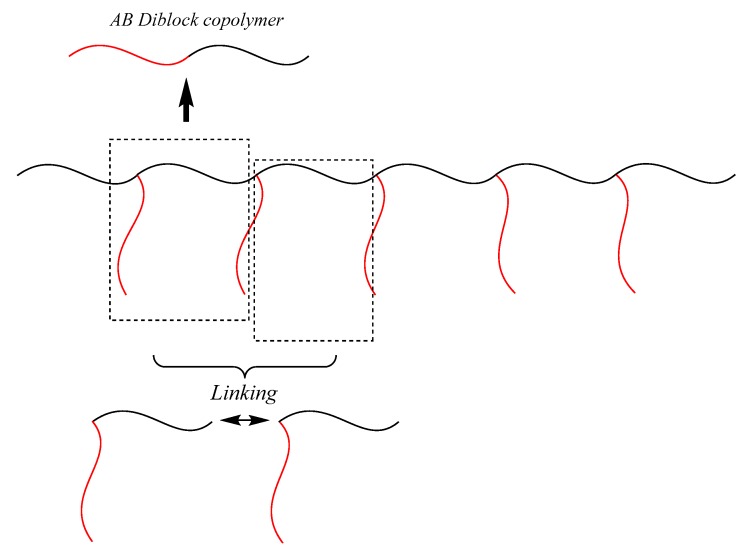
Synthesis of (EDGP) by the linking reaction between two AB diblock copolymers.

It is considered that the iterative methodologies are applicable because (EDGP)s comprise several repeating units linked each other. As can be seen in Figure 4, one building unit of graft polymer corresponds to an AB diblock copolymer and the two block copolymers are linked between the chain-end and in-chain of diblock copolymers to make the graft unit. Therefore, if the linking of diblock copolymers can be iterated, a series of exactly defined graft copolymers would be synthesized.

**Scheme 8:**
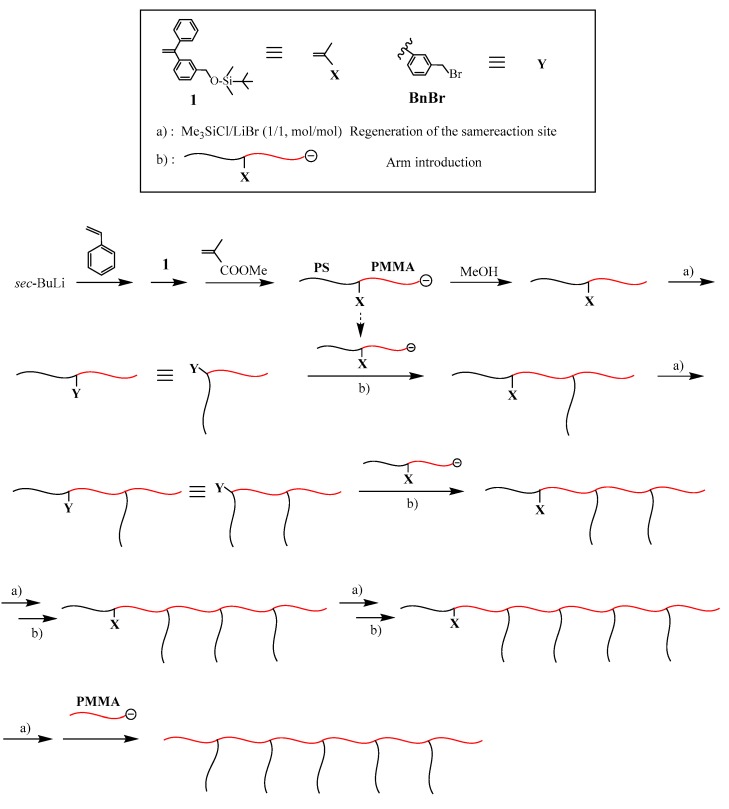
Successive synthesis of exactly defined (PMMA–*graft*–PSt)s by the iterative methodology. Reproduced with permission from reference [93]. Copyright 2009 American Chemical Society.

On the basis of the linking manner indicated in Figure 4, we proposed the iterative methodology as illustrated in Scheme 8 [93,94]. This methodology is basically the same as those shown in Scheme 1, Scheme 4, Scheme 5 and Scheme 6, except for the use of the living in-chain-**X**-functionalized diblock copolymer as the building block. It was prepared by the sequential addition of styrene, **1**, and MMA and, after the termination, the TBDMS ether was converted to the BnBr reaction site. Another living in-chain-TBDMS ether-functionalized PSt–*block*–PMMA was reacted with the resulting PA-functionalized PSt–*block*–PMMA to link the two block copolymers between the chain-end and the in-chain. Since the TBDMS ether was also introduced, repetition of the same reaction sequence is possible. In practice, the reaction sequence was iterated four more times. The final polymer with five graft chains was yielded by linking the intermediate graft copolymer with a living (PMMA). The requisite structures of the synthesized graft copolymers all were clearly evidenced from the results summarized in Table 6. Thus, the proposed methodology satisfactorily worked as expected.

Each reaction sequence involves the linking reaction between two block copolymers and the conversion to the reaction site, which are equal to the “polymer chain introduction” and “regeneration of the same reaction site” and can be iterated via the regenerated same reaction site. The three parameters of the resulting polymers are controlled and can be intentionally changed by the living first and second blocks. With this in mind, three additional acid-labile segment containing exactly defined P^t^BMA–*graft*–PSt, poly(ferrocenylmethyl methacrylate (PFMMA))–*graft*–PSt, and P2VPy–*graft*–PSt were also synthesized by the same iterative methodology using a PA reaction site. All of these polymers thus synthesized by the iterative methodology are the first successful (EDGP)s with more than three graft chains. 

**Scheme 9:**
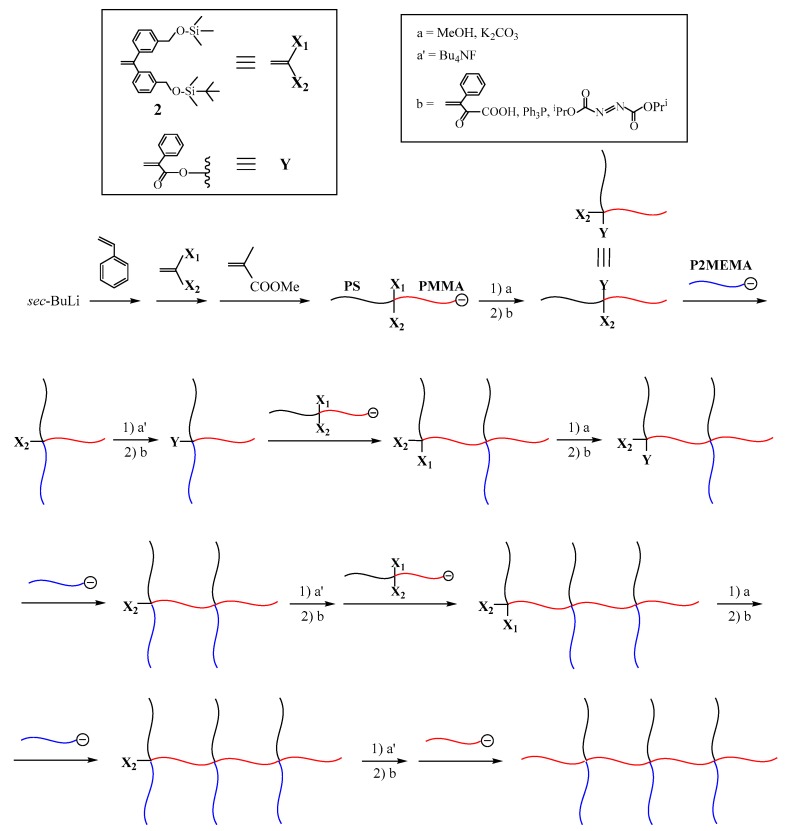
Successive synthesis of EDG terpolymers by the iterative methodology with the use of living in-chain-(**X_1_** and **X_2_**)-functionalized diblock copolymer. Reproduced from Reference [97] with permission from the Centre National de la Recherche Scientifique (CNRS) and The Royal Society of Chemistry.

Recently, we have succeeded in the synthesis of an EDG terpolymer with two different graft chains per one graft point by the iterative methodology using a living in-chain-(**X_1_** and **X_2_**)-functionalized diblock copolymer (see Scheme 9) [97]. Both the **X_1_** and **X_2_** functionalities are designed so as to be converted in turn to the first and second **Y** reaction sites in different reaction steps. The first graft chain corresponding to the first PSt block was already present. The second graft chain was introduced by linking a living polymer at the first **Y** reaction site converted from the **X_1_** function. Next, a newly prepared living in-chain-(**X_1_** and **X_2_**)-functionalized block copolymer reacted with the second **Y** reaction site from the **X_2_** function. As the **X_1_** and **X_2_** functionalities were thus reintroduced, the same reaction sequence was iterated to continue the synthesis of EDG terpolymers.

As mentioned before, the TMS and TBDMS ethers are used as the **X_1_** and **X_2_** functionalities, respectively. In the first reaction sequence, a living in-chain-(TMS and TBDMS ethers)-functionalized PSt–*block*–PMMA was prepared by the sequential polymerization of styrene, **2**, and MMA. The introduced TMS group in-chain was deprotected with MeOH containing K_2_CO_3_ and the regenerated OH group was converted to the PA reaction site. The second graft chain was introduced by linking with a living poly(2-methoxyethyl methacrylate) (P2MEMA). The TBDMS group was then deprotected with (C_4_H_9_)_4_NF, followed by converting to the reaction site. A living in-chain-(TMS and TBDMS ethers)-functionalized PSt–*block*–PMMA was newly prepared and reacted with the resulting PA-functionalized 3-arm (μ-SP) composed of PSt, (PMMA), and P2MEMA. By this linking reaction, one branched unit was made and the TMS and TBDMS ethers were also reintroduced. Repetition of the reaction sequence is possible via the two silyl ethers. An EDG terpolymer possessing three branched units was yielded after iterating the reaction sequence two more times, followed by linking with a living (PMMA) in the final step. Since all of the repeating reaction sequences proceeded almost quantitatively, the target polymers were always obtained in ca. 100% yields. 

The polymers all were obtained with well-controlled *M*_n_ values and compositions (see Table 7). Accordingly, the proposed methodology also effectively worked to successfully yield the target EDG terpolymers. The three structural parameters of such graft terpolymers are also controlled and intentionally changed by the living polymerization in each step. 

**Scheme 10:**
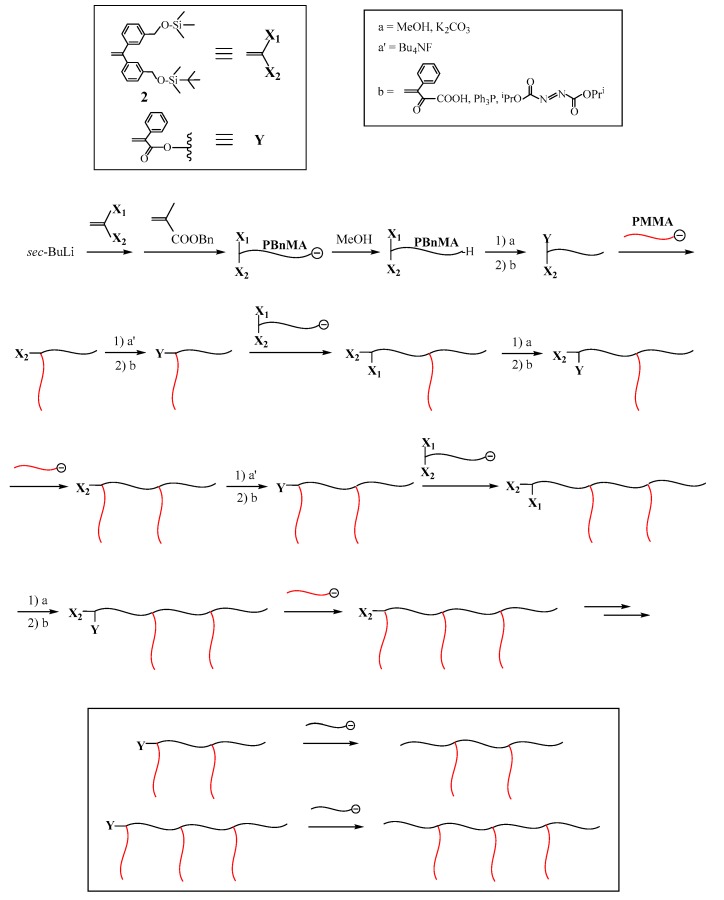
Successive synthesis of (EDGP)s by the iterative methodology with the use of living α-chain-end-(**X_1_** and **X_2_**)-functionalized polymer. Reproduced with permission from reference [96]. Copyright 2015 American Chemical Society.

With the iterative methodology shown in Scheme 8, both graft and main chains are advantageously introduced at the same time. We have recently developed an alternative methodology using a living α-chain-end-(**X_1_** and **X_2_**)-functionalized polymer (see Scheme 10) [96]. The same as Scheme 9, the **X_1_** and **X_2_** functionalities are TMS and TBDMS ethers and sequentially deprotected one by one to convert to the first and second PA (**Y**) reaction sites. The synthesis was started to prepare a living α-chain-end-(TMS and TBDMS ethers)-functionalized poly(benzyl methacrylate) (PBnMA) by the living polymerization of BnMA with the functional initiator obtained from *sec*-BuLi and **2**. After the termination and the conversion of the TMS ether to the reaction site, a living (PMMA) reacted with the reaction site to result in an in-chain-TBDMS ether-functionalized (PMMA)–*block*–PBnMA. The TBDMS ether was then converted to the reaction site. The in-chain-PA-functionalized (PMMA)–*block*–PBnMA thus prepared was linked with a newly prepared living α-chain-end-(TMS and TBDMS ethers)-functionalized PBnMA. By this reaction, one branched unit was made and the TMS and TBDMS ethers were reintroduced. The reaction sequence was iterated twice. In each of the intermediate polymers thus obtained, the TBDMS ether was converted to the reaction site, followed by linking with a living PBnMA, to synthesize the exactly defined PBnMA–*graft*–PMMA with two and three (PMMA) graft chains. 

The results summarized in Table 8 clearly show the successful synthesis of the exactly defined (PBnMA–*graft*–PMMA)s. Thus, it is possible to synthesize all poly(methacrylate)-based (EDGP)s by this methodology. Needless to say, the synthesis of such (EDGP)s is difficult by the methodology shown in Scheme 8. Similarly, a series of exactly defined (PBnMA–*graft*–P2VPy)s were synthesized by the same methodology, in which living P2VPy was used instead of living (PMMA) in each reaction sequence. 

**Scheme 11:**
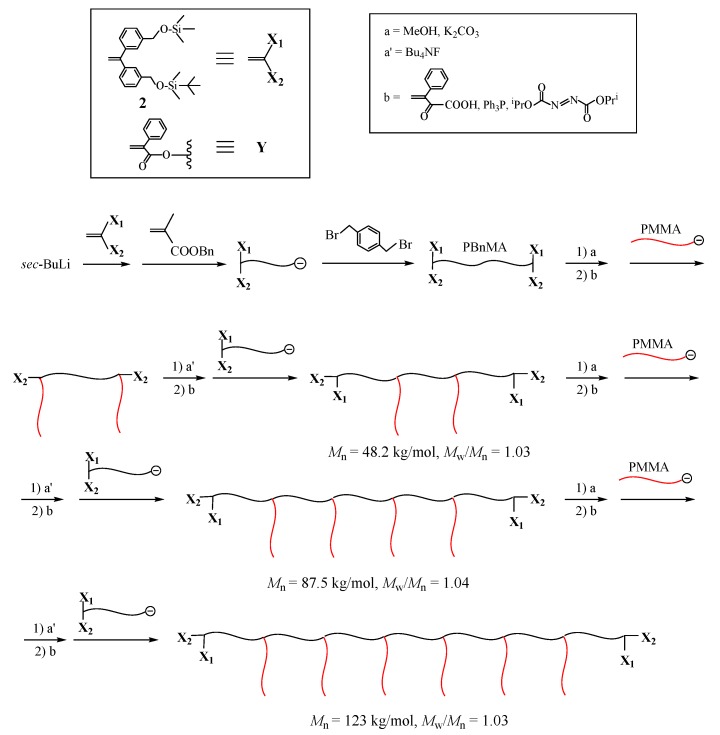
Successive synthesis of exactly defined (PBnMA–*graft*–PMMA)s with two, four, and six (PMMA) graft chains by the iterative methodology using difunctional PBnMA as the starting polymer.

A difunctional PBnMA with the TMS and TBDMS termini was prepared by the coupling reaction of a living α-chain-end-(TMS and TBDMS ethers)-functionalized PBnMA with *p*,*p*’-xylene dibromide. When this polymer was used as the starting polymer, (EDGP)s with two, four, and six (PMMA) graft chains were readily synthesized with less repetition of the reaction sequence (only three times) (see Scheme 11).

In summary, we demonstrated the successful syntheses of EDG co- and terpolymers by developing the three iterative methodologies shown in Scheme 8, Scheme 9 and Scheme 10. As often suggested in these methodologies, they are basically the same as the methodologies for the synthesis of (μ-SP)s and (DHBP)s. Three different building blocks, i.e., living in-chain-**X**-functionalized diblock copolymer, living in-chain-(**X_1_** and **X_2_**)-functionalized diblock copolymer, and living α-chain-end-(**X_1_** and **X_2_**)-functionalized polymer, were employed. It should also be noted that the TBDMS ether (**X**), TMS ether (**X_1_**), and TBDMS ether (**X_2_**) functionalities and the BnBr or PA (**Y**) reaction site are the same as those used in the previous methodologies.

### 2.4. Block Polymers ((BP)s)

Although (BP)s are not generally categorized in macromolecular architectures, multiblock polymers with more than three blocks are added to macromolecular architectures in this article because they are composed of several polymer segments or repeating units linked in a line. In general, well-defined (BP)s are synthesized by the sequential addition of different monomers to an appropriate anionic initiator, so-called “sequential block polymerization” [98]. Using two monomers with similar reactivities, all possible (BP)s are readily obtained without difficulty because the crossover polymerization is acceptable among these monomers. In fact, AB, ABA, BAB, and (AB)*_n_* multiblock copolymers are synthesized by the sequential block polymerization. In the case using three monomers, the syntheses of ABC, ACB, and BAC triblock terpolymers are achieved by sequentially adding the corresponding monomers to the initiator. 

In contrast, when monomers with different reactivities are used, the synthetically feasible (BP)s are considerably restricted because the crossover polymerization is difficult. Among these monomers, a less reactive chain-end anion is always produced from a more reactive monomer and vice-versa. This often causes a serious problem that a less reactive chain-end anion is not able to polymerize a less reactive monomer. As a result, no block copolymer is formed. In this section, the synthetic difficulty of (BP)s using monomers with different reactivities and how to overcome such the difficulty will be described [1,5,16,17,99,100,101,102,103,104].

#### 2.4.1. Synthetic Possibility of Triblock Co- and Terpolymers

In order to examine the synthetic possibility of triblock copolymer using two monomers with different reactivities, styrene and MMA are chosen. As is known, MMA is more reactive (or electrophilic) than styrene because of the stronger electron-withdrawing character of the carbonyl group to reduce the electron-density of the vinyl group. In contrast, the (PMMA) anion becomes less reactive (or nucleophilic) than that of PSt by the presence of the same stronger electron-withdrawing carbonyl group, which reduces electron-density of the anion. Accordingly, (PMMA) anion cannot polymerize less reactive styrene under the normal conditions, while MMA is readily polymerized with more reactive PSt anion. This means that (PMMA)–*block*–PSt cannot be obtained by the sequential addition of first MMA and styrene. On the other hand, less reactive styrene is first polymerized to produce the more reactive PSt anion, followed by addition of more reactive MMA, yielding PSt–*block*–PMMA. Thus, the monomer addition order is very critical. Due to the same reactivity problem, neither triblock copolymers of PSt–*block*–PMMA–*block*–PSt and (PMMA)–*block*–PSt–*block*–PMMA nor multiblock copolymers of PSt–*block*–PMMA–*block*–PSt–*block*–PMMA…; i.e., (PSt–*block*–PMMA)*_n_* can be synthesized. Thus, the synthesis of all possible (BP)s, except for PSt–*block*–PMMA, is difficult by the sequential block copolymerization.

**Scheme 12:**
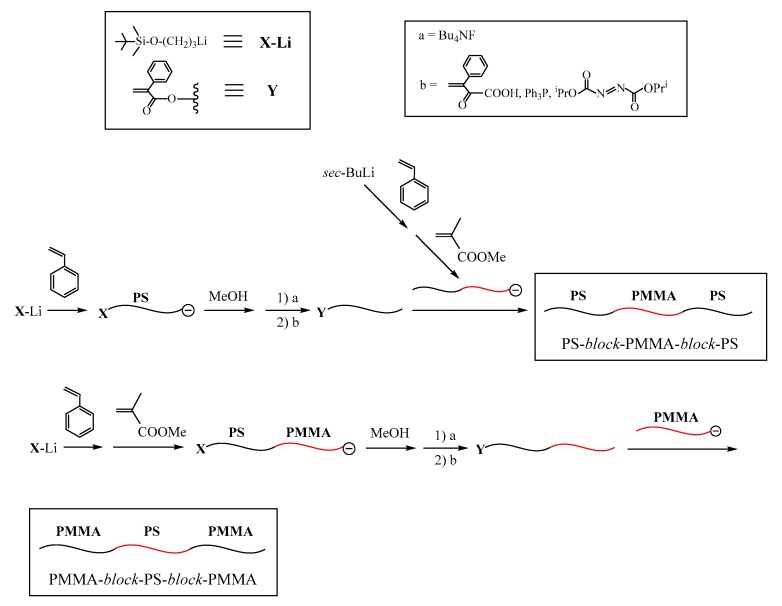
Synthesis of PSt–*block*–PMMA–*block*–PSt and (PMMA)–*block*–PSt–*block*–PMMA by a new methodology combining living sequential block copolymerization with a 1:1 addition reaction. Reproduced with permission from reference [100]. Copyright 2011 American Chemical Society.

To overcome such synthetic difficulty, we have recently proposed a more general methodology combining living anionic sequential block copolymerization with a 1:1 addition reaction as illustrated in Scheme 12 [100,101,102]. For the synthesis of PSt–*block*–PMMA–*block*–PSt, an α-chain-end-**X**-functionalized PSt was first prepared by the living polymerization of styrene with an **X**-functionalized initiator. The introduced **X** α-terminus was then converted to a **Y** reaction site. A living PSt–*block*–PMMA was prepared by the sequential polymerization and reacted with the α-chain-end-**Y**-functionalized PSt, yielding the target PSt–*block*–PMMA–*block*–PSt. A (PMMA)–*block*–PSt–*block*–PMMA with the opposite sequence was synthesized by the linking of a living (PMMA) with an α-chain-end-**Y**-functionalized PSt–*block*–PMMA. Here, the **X** and **Y** functionalities are TBDMS ether and PA reaction site, respectively, similar to several schemes shown in this article. Furthermore, synthetically difficult PSt–*block*–P2VPy–*block*–PSt, P2VPy–*block*–PSt–*block*–P2VPy, P2VPy–*block*–PMMA–*block*–P2VPy, and (PMMA)–*block*–P2VPy–*block*–PMMA were also obtained by the same methodology. 

Among the above synthesized (BCP)s, P2VPy–*block*–PSt–*block*–P2VPy, (PMMA)–*block*–PSt–*block*–PMMA, and (PMMA)–*block*–P2VPy–*block*–PMMA can be synthesized by the sequential addition of styrene or 2VPy and either 2VPy or MMA to an appropriate difunctional initiator. In these polymers, the P2VPy or (PMMA) both side blocks are always equal in molecular weight. Very interestingly and importantly, the proposed methodology enables to synthesize the triblock copolymers with molecular weight-asymmetry in both side blocks because the molecular weight of each side block can be changed by the living anionic polymerization or sequential block copolymerization prior to the linking reaction. The following polymers were such examples: P2VPy–*block*–PSt–*block*–P2VPy (*M*_n_ = 19.5/10.0/10.4 kg·mol^−1^), (PMMA)–*block*–PSt–*block*–PMMA (*M*_n_ = 18.7/11.2/11.6 kg·mol^−1^), and (PMMA)–*block*–P2VPy–*block*–PMMA (*M*_n_ = 6.30/12.6/20.5 kg·mol^−1^) [100]. 

The next question is the synthetic possibility of triblock terpolymer using three monomers with different reactivities. In order to understand the synthetic situation, styrene, 2VPy, and MMA were used as the three monomers. The monomer reactivity increases from styrene, 2VPy, to MMA in this order, while the reactivity of chain-end anion decreases from living PSt (hereinafter abbreviated as PSt^−^), P2VPy^−^, to (PMMA)^−^. Their relationships in the polymerization are listed in Table 9. The most reactive PSt^−^ initiates the polymerization of 2VPy and MMA, which are more reactive than styrene, while neither styrene nor 2VPy is polymerized with the least reactive (PMMA)^−^. The 2VPy^−^ with the reactivity between PSt^−^ and (PMMA)^−^ polymerizes more reactive MMA, but can polymerize less reactive styrene very sluggishly along with the unwanted side reactions during the polymerization. Based on such the relationships, it is understandable that the ABC triblock terpolymer of PSt–*block*–P2VPy–*block*–PMMA can be synthesized, but the syntheses of PSt–*block*–PMMA–*block*–P2VPy (ACB) and P2VPy–*block*–PSt–*block*–PMMA (BAC) are difficult by the sequential block terpolymerization due to the monomer and anion reactivity mismatch [105,106,107,108,109].

**Scheme 13:**
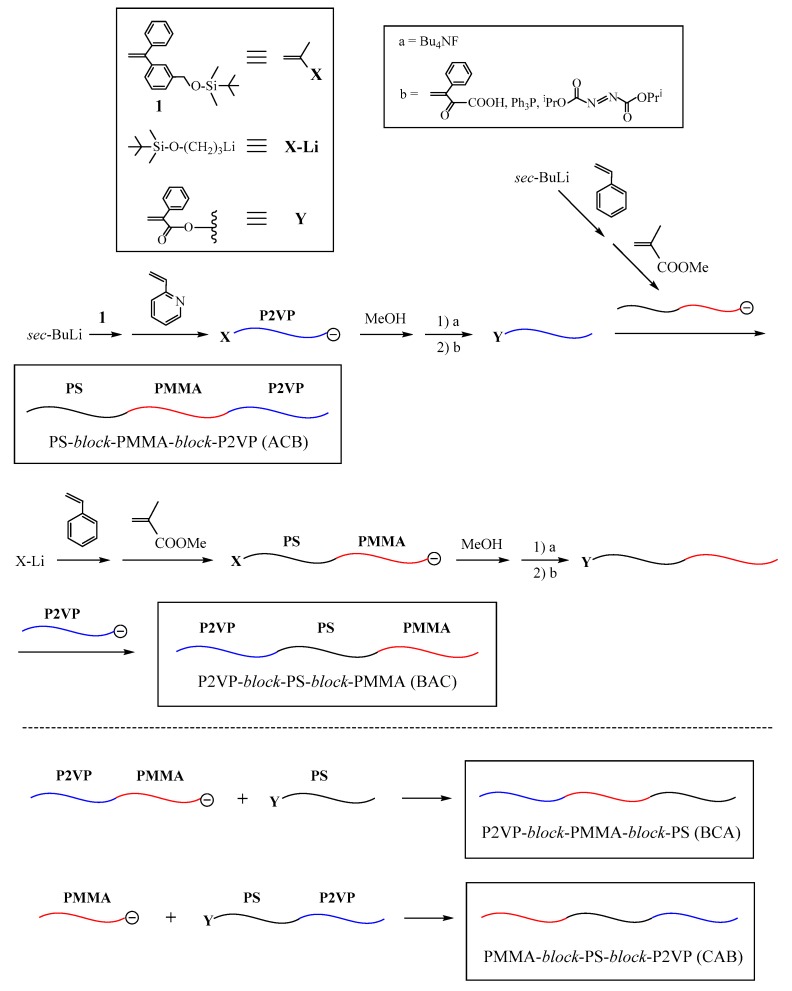
Synthesis of ACB and BAC as well as BCA and CAB triblock terpolymers by the methodology combining linking chemistry with living sequential block copolymerization. Reproduced with permission from reference [100]. Copyright 2011 American Chemical Society.

The methodology combining living sequential block copolymerization with a 1:1 addition reaction useful for triblock copolymers synthesis was also effective for the synthesis of the ACB and BAC triblock terpolymers (see Scheme 13) [100]. To synthesize the PSt–*block*–PMMA–*block*–P2VPy (ACB), a living PSt–*block*–PMMA was first prepared and in situ reacted with an α-chain-end-PA-functionalized P2VPy. Similarly, the P2VPy–*block*–PSt–*block*–PMMA (BAC) could be synthesized by the linking of a living P2VPy with an α-chain-end-PA-functionalized PSt–*block*–PMMA.

Both P2VPy–*block*–PMMA–*block*–PSt (BCA) and (PMMA)–*block*–PSt–*block*–P2VPy (CAB) are considered to be the same in structure as the ACB and BAC. However, another synthetic design is possible for these polymers (see also Scheme 13). A P2VPy–*block*–PMMA–*block*–PSt (BCA) was synthesized by the linking of a living P2VPy-*block*-PMMA with a PSt with the PA α-terminus. Similarly, a (PMMA)–*block*–PSt–*block*–P2VPy (CAB) was obtained by the reaction of a living (PMMA) with an α-chain-end-PA-functionalized PSt-*block*-P2VPy. Their requisite structures were evident (see Table 10) [100]. 

Using monomers with different reactivities, the synthesis of all possible triblock co- and terpolymers, except for the ABC type, is difficult by the sequential block polymerization because of the mismatch among monomer electrophilicities and chain-end anion nucleophilicities. To overcome the difficulty, the new methodology combining living sequential block copolymerization with a 1:1 addition reaction has been proposed and found to allow access to such synthetically difficult triblock co- and terpolymers without consideration about the monomer and anion reactivities.

Previously, several research groups also reported the efficient methodologies to overcome the synthetic difficulty of block polymers using monomers with different reactivities [110,111,112,113,114,115,116,117,118,119,120]. It is believed that the present methodology herein proposed seems to be more general in synthesis than those previous methodologies. The related block polymers can also be synthesized by the living/controlled radical polymerization or the combination of living/controlled radical polymerization with Click reactions. However, the resulting block polymers always possess relatively broad molecular weight distributions [121,122,123,124,125,126].

#### 2.4.2. Multiblock Copolymer Synthesis

**Scheme 14:**
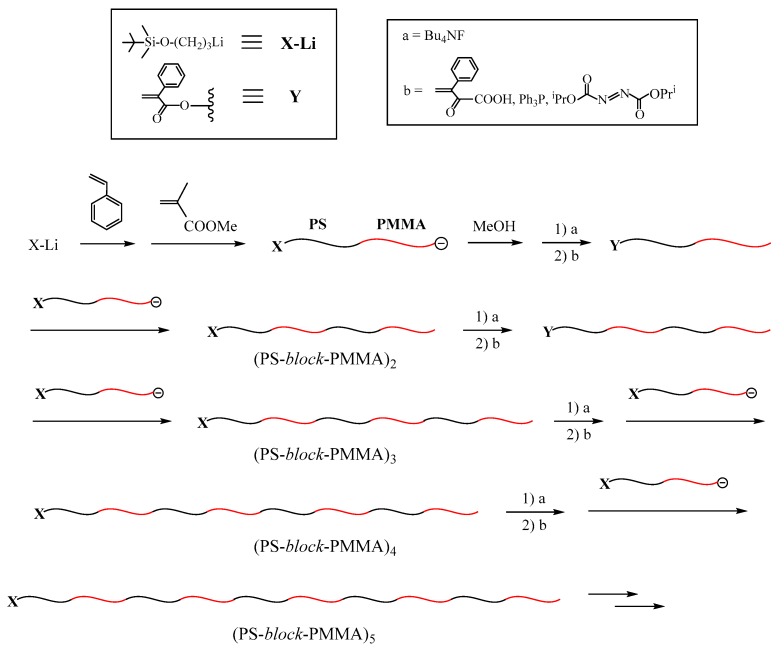
Synthesis of (PS–*block*–PMMA)*_n_* (*n* = 1–5) by the iterative methodology. Reproduced with permission from reference [99]. Copyright 2011 American Chemical Society.

The methodology shown in Scheme 12 was extended to the iterative methodology to synthesize multiblock copolymers of (PSt–*block*–PMMA)*_n_* (see Scheme 14) [99]. In this methodology, a living α-chain-end-TBDMS ether-functionalized PSt–*block*–PMMA is utilized as the building block to simultaneously introduce both PSt and (PMMA) blocks. In the first sequence, the living α-chain-end-TBDMS ether-functionalized PSt–*block*–PMMA was prepared and, after the termination, the introduced TBDMS ether was converted to the PA reaction site. The PA-functionalized PSt–*block*–PMMA was linked with a newly prepared living α-chain-TBDMS ether-functionalized PSt–*block*–PMMA to yield an ABAB tetrablock copolymer of PSt–*block*–PMMA–*block*–PSt–*block*–PMMA ((PSt–*block*–PMMA)_2_). Since the TBDMS ether α-terminus is present, the reaction sequence is possible to be iterated. Indeed, the reaction sequence was further iterated to synthesize a hexablock, octablock, and even decablock copolymer of (PSt–*block*–PMMA)_5_. 

As shown in Table 11, the resulting multiblock copolymers all possessed precisely controlled *M*_n_ values and compositions, and low polydispersity indexes. Thus, requisite and well-defined multiblock copolymers up to a decablock type were successfully synthesized. As every reaction sequence proceeded almost quantitatively, all of the multiblock copolymers were obtained in ca. 100% yields. As the final (PSt–*block*–PMMA)_5_ still possesses the TBDMS ether α-terminus, the reaction sequence can be further iterated. Two more multiblock copolymers comprising PSt and either (P^t^BMA) or P2VPy were also synthesized (see also Table 11) [99]. The linking reaction between the two diblock copolymers and conversion to the reaction site in each sequence are equal to the “polymer chain introduction” and “regeneration of the reaction site” steps. Thus, the iterative methodology herein developed also effectively worked for the synthesis of multiblock copolymers.

**Scheme 15:**
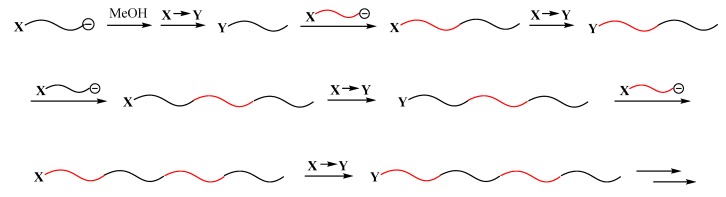
Synthesis of multiblock copolymers by the iterative methodology using a living α-chain-end-**X**-functionalized polymer.

As illustrated in Scheme 15, the same multiblock copolymers can be obtained by the similar methodology using a living α-chain-end-**X**-functionalized polymer as the building block. However, the use of living α-chain-end-**X**-functionalized diblock copolymer is much more preferable because of the reaction step reduction. The synthesis of (ABC)*_n_* multiblock terpolymers may be feasible by the methodology with the use of living α-chain-end-**X**-functionalized ABC triblock terpolymer and is now under study.

It is believed that the syntheses of triblock copolymers, triblock terpolymers except for the ABC type, and multiblock copolymers are difficult by the direct sequential block co- and terpolymerization using monomers with different reactivities. The methodology combining living sequential block copolymerization with a 1:1 addition reaction and the extended iterative methodology have demonstrated the useful means to successfully synthesize such (BP)s difficult by sequential block polymerization. 

## 3. Concluding Remarks and Future Outlook

Throughout this article, we have demonstrated the successful development of a novel all-around iterative methodology for the syntheses of multicomponent (μ-SP)s, high generation (DHBP)s, (EDGP)s, and multiblock polymers with more than three blocks. In this methodology, two reaction steps, i.e., the polymer chain introduction and the regeneration of the same reaction site, are involved in each reaction sequence and iterated to construct the above-mentioned polymers. The following key building block is individually designed and used on the basis of their architectures: living chain-end-**X**-functionalized polymer (Scheme 1), living α-chain-end-((**X**)**_2_** or (**X**)**_4_**)-functionalized polymer (Scheme 4, Scheme 5 and Scheme 6), living in-chain-**X**-functionalized diblock copolymer (Scheme 8), living in-chain-(**X_1_** and **X_2_**)-functionalized diblock copolymer (Scheme 9), living α-chain-end-(**X_1_** and **X_2_**)-functionalized polymer (Scheme 10), and living α-chain-end-**X**-functionalized diblock copolymer (Scheme 14). The **X**, **X_1_**, and **X_2_** functionalities are TBDMS ether, TMS ether, and TBDMS ether convertible to the BnBr or PA reaction site. In certain cases, the functional anions with TMS (**X_1_**), TBDMS (**X_2_**), and THP ethers (**X_3_**) are employed (Scheme 2 and Scheme 3) instead of living functionalized polymers. One more important advantage of this methodology is that the use of complicated multistep selective reactions and reaction site with different reactivities generally required for macromolecular architecture synthesis are completely avoided because the polymer segment(s) are introduced one by one in each reaction step. 

As often mentioned, all the iterative methodologies herein developed are basically the same in concept as the all-around iterative methodology mentioned in introduction. As the future synthetic potential, it is expected that different key building blocks can be employed together in the same methodology to result in the synthesis of more complex macromolecular architectures with mixed structures. Thus, synthetic limitation and difficulty of macromolecular architectures have been greatly surpassed with the progress of the iterative methodology.

## Figures and Tables

**Table 1:** Characterization results of (μ-SP)s synthesized by the iterative methodology.

Polymer	*M*_n_ (kg/mol)		*M*_w_/*M*_n_	Composition (wt % wt % wt %)	
	Calcd.	RALLS ^a^	SEC	Calcd.	^1^H NMR
A	10.4	10.0	1.02	100	100
AB	19.8	20.8	1.02	50/50	48/52
3-arm ABC	31.7	34.4	1.02	32/34/34	34/32/34
4-arm ABCD ^b^	45.8	46.5	1.02	26/24/25/25	27/23/25/25

^a^ Determined by SEC with RI, viscometer, and right angle laser light scattering (RALLS) detector. ^b^ A, B, C, and D arms correspond to polystyrene, poly(α-methylstyrene), poly(4-methylstyrene), and poly(methyl methacrylate), respectively.

**Table 2:** Characterization results of (μ-SP)s synthesized by the iterative methodology using **X_1_** and **X_2_** functionalities.

Polymer	*M*_n_ (kg/mol)		*M*_w_/*M*_n_	Composition (wt % wt % wt %)	
	Calcd.	RALLS ^a^	SEC	Calcd.	^1^H NMR
3-arm ABC	32.2	33.8	1.03	38/31/31	37/32/31
4-arm ABCD	43.4	43.0	1.03	30/25/22/23	36/26/20/24
5-arm ABCDE ^b^	52.0	51.5	1.03	24/21/18/19/18	26/20/16/20/18

^a^ Determined by SEC with RI, viscometer, and right angle laser light scattering (RALLS) detector. ^b^ A, B, C, D, and E arms correspond to (PMMA), poly(ethylmethacrylate), poly(*tert*-butylmethacrylate), poly(benzylmethacrylate), and poly(2-methoxyethylmethacrylate), respectively.

**Table 3:** Characterization results of (μ-SP)s synthesized by the iterative methodology using **X_1_**, **X_2_**, and **X_3_** functionalities.

Polymer	*M*_n_ (kg/mol)		*M*_w_/*M*_n_	Composition (wt % wt % wt %)	
	Calcd.	RALLS	SEC	Calcd.	^1^H NMR
ABC	35.0	34.9	1.03	30/35/35	31/35/34
ABCD	45.9	47.1	1.03	23/26/27/25	24/25/27/24
ABCDE	56.6	53.5	1.04	20/21/22/20/17	20/23/21/20/16
ABCDEF	62.8	64.9	1.05	17/18/19/17/15/14	18/20/18/17/14/13
ABCDEFG ^a^	73.8	74.2	1.04	14/16/17/15/13/13/12	16/17/16/14/12/12/13

^a^ A, B, C, D, E, F, and G arms correspond to poly(cyclohexylmethacrylate), PSt, poly(4-methoxystyrene), poly(4-methylstyrene), (PMMA), poly(ethylmethacrylate), and poly(2-methoxyethylmethacrylate), respectively.

**Table 4:** Characterization results of the DHB (PMMA)s (1G–7G).

Polymer	*M*_n_ (kg/mol)		*M*_w_ (kg/mol)		*M*_w_/*M*_n_	End-Functionality	
	Calcd.	^1^H NMR	Calcd.	SLS ^a^	SEC	Calcd.	^1^H NMR
1G	14.6	14.2	14.9	14.8	1.02	8	7.90
2G	43.0	42.9	43.9	44.2	1.02	16	16.0
3G	97.3	98.4	99.2	105	1.02	32	32.6
4G	219	219	223	230	1.02	64	64.0
5G	452	449	462	472	1.03	128	127
6G	980	974	1000	1060	1.02	256	254
7G	1950	1940	1980	1970	1.02	512	509

^a^ static light scattering (SLS). Arm segments are adusted to be in the range of 3500~4000 g·mol^−1^ in *M*_n_ value.

**Table 5:** Characterization results of highly dense DHB (PMMA)s (1G–4G).

Polymer	*M*_n_ (kg/mol)			*M*_w_/*M*_n_	Composition (wt % wt % wt %)	
	Calcd.	^1^H NMR	SLS	SEC	Calcd.	^1^H NMR
1G	52.8	52.2	51.6	1.02	16	16.1
2G	236	234	247	1.02	64	63.6
3G	938	959	930	1.01	256	259
4G-1	2350	2330	2330	1.02	512	521
4G-2	1860	1900	1860	1.03	512	528
4G-3	1910	1930	1880	1.02	512	516

PMMA arm segments are adjusted to be ca. 10,000 g·mol^−1^ in *M*_n_ value.

**Table 6:** Characterization results of exactly defined (PMMA–*graft*–PSt)s synthesized by the iterative methodology.

Polymer	*M*_n_ (kg/mol)			*M*_w_/*M*_n_	Composition (wt % wt % wt %) ^a)^	
	Calcd.	^1^H NMR	RALLS	SEC	Calcd.	^1^H NMR
AB Diblock	12.5	12.6	12.6	1.03	50/50	48/52
EG-2 ^b)^	22.2	22.5	23.6	1.02	51/49	50/50
EG-3 ^c)^	33.8	35.4	34.6	1.04	50/50	49/51
EG-4 ^d)^	43.5	46.1	45.6	1.04	49/51	50/50
EG-5 ^e)^	56.2	56.0	55.0	1.02	45/55	45/55

^a)^ PS/PMMA.

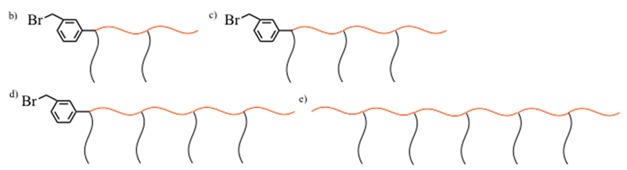

**Table 7:** Characterization results of EDG terpolymers synthesized by the iterative methodology.

Polymer	*M*_n_ (kg/mol)		*M*_w_/*M*_n_	Composition (wt % wt % wt %) ^a)^	
	Calcd.	RALLS	SEC	Calcd.	^1^H NMR
AB Diblock	20.9	21.0	1.03	48/52/0	48/52/0
EGTP-1′ ^b)^	29.1	30.2	1.02	35/37/28	34/38/28
EGTP-2′ ^c)^	61.7	61.5	1.04	34/35/31	32/38/30
EGTP-3′ ^d)^	87.8	87.1	1.04	33/34/33	31/33/35
EGTP-3 ^e)^	97.4	97.0	1.02	39/31/30	37/32/31

^a)^ (PMMA)/PS/P2MEMA.

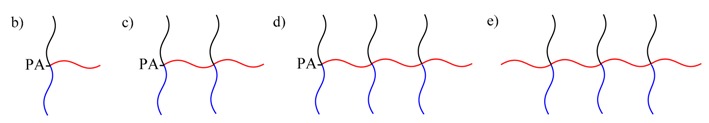

**Table 8:** Characterization results of (EDGP)s by the iterative methodology.

Polymer	*M*_n_ (kg/mol)		*M*_w_/*M*_n_	Composition (wt % wt % wt %) ^a)^	
	Calcd.	RALLS	SEC	Calcd.	^1^H NMR
EGTP-1′ ^b)^	19.7	18.7	1.02	40/60	39/61
EGTP-1 ^c)^	30.1	28.4	1.02	60/40	60/40
EGTP-2′ ^d)^	39.3	39.1	1.02	45/55	44/56
EGTP-2 ^e)^	49.6	49.5	1.02	56/44	55/45
EGTP-3′ ^f)^	59.9	60.9	1.03	47/53	47/53
EGTP-3 ^g)^	72.7	71.3	1.03	55/45	56/44

^a)^ Determined by SEC with RI, viscometer, and right angle laser light scattering (RALLS) detector. ^b)^ APBnMA/PMMA.

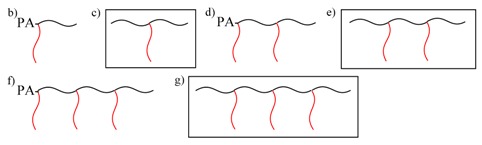

**Table 9:** Relationships among monomers and chain-end anions in polymerization.

	Monomer	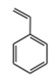	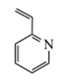	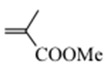
Growing Chain-End Anion				
PSt^−^		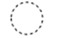	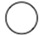	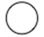
(P2VPy)^−^		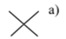	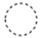	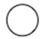
(PMMA)^−^		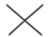	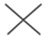	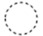

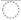
: Homopolymerization. 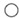: Living polymerization. 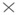: No polymerization. ^a)^ The polymerization proceeds very sluggishly along with unwanted side reacions.

**Table 10:** Characterization results of ABC, ACB, BAC, BCA, and CAB triblock terpolymers.

Polymer	*M*_n_ (kg/mol)		*M*_w_/*M*_n_	Composition (wt % wt % wt %)	
	Calcd.	RALLS	SEC	Calcd.	^1^H NMR
ABC	40.5	40.0	1.04	33/34/33	32/35/33
ACB	32.2	34.6	1.04	25/58/17	27/54/19
BAC	28.9	30.1	1.05	32/34/34	31/34/35
BCA	37.2	38.2	1.04	32/35/33	31/37/32
CAB	34.0	34.3	1.04	31/33/36	29/37/34

A, B and C blocks are PSt, P2VPy, and (PMMA), respectively.

**Table 11:** Characterization results of (PS–*block*–PMMA)*_n_*, (PS–*block*–P^t^BMA)*_n_* and (PS–*block*–P2VP)*_n_* synthesized by the iterative methodology.

Polymer	*M*_n_ (kg/mol)		*M*_w_/*M*_n_	Composition (wt % wt % wt %)	
	Calcd.	RALLS	SEC	Calcd.	^1^H NMR
PS–*b*–PMMA	10.5	11.4	1.03	50/50	48/52
(PS–*b*–PMMA)_2_	26.4	28.2	1.03	46/54	47/53
(PS–*b*–PMMA)_3_	37.0	40.5	1.03	45/55	43/57
(PS–*b*–PMMA)_4_	51.0	53.6	1.04	45/55	43/57
(PS–*b*–PMMA)_5_	64.5	66.4	1.06	45/55	45/55
(PS–*b*–P^t^BMA)_2_	24.0	25.8	1.03	47/53	46/54
(PS–*b*–P^t^BMA)_3_	41.5	46.6	1.06	48/52	46/54
(PS–*b*–P2VP)_2_	24.3	25.3	1.04	49/51	49/51
(PS–*b*–P2VP)_3_	37.2	38.3	1.03	49/51	48/52

## References

[1] Hadjichristidis N., Pitsikalis M., Pispas S., Iatrou H. (2001). Polymers with Complex Architecture by Living Anionic Polymerization. Chem. Rev..

[2] Hadjichristidis N., Pitsikalis M., Pispas S., Mays J.W. (2006). Macromolecular Architectures by Living and Controlled/Living Polymerizations. Prog. Polym. Sci..

[3] Polymeropoulos G., Zapsas G., Ntetsikas K., Bilalis P., Gnanou Y., Hadjichristidis N. (2017). Polymers with Complex Architectures. Macromolecules.

[4] Lee M., Cho B.-K., Zin W.-C. (2001). Supramolecular Structures from Rod-Coil Block Copolymers. Chem. Rev..

[5] Hadjichristidis N., Iatrou H., Pitsikalis M., Pispas S., Avgeropoulos A. (2005). Linear and Non-Linear Triblock Terpolymers. Synthesis, Self-Assembly in Selective Solvents and in Bulk. Prog. Polym. Sci..

[6] Li Z., Hillmyer M.A., Lodge T.P. (2006). Control of Structure in Multicompartment Micelles by Blending μ-ABC Star Terpolymers with AB Diblock Copolymers. Macromolecules.

[7] Gao H., Matyjaszewski K. (2009). Synthesis of Functional Polymers with Controlled Architecture by CRP of Monomers in the Presence of Cross-Linkers: From Stars to Gels. Prog. Polym. Sci..

[8] Matsushita Y., Hayashida K., Takano A. (2010). Jewelry Box of Morhologies with Mesoscopic Length Scale-ABC Star-shaped Terpolymers. Macromol. Rapid Commun..

[9] Kim H.-C., Park S.-M., Hinsberg W.D. (2010). Block Copolymer Based Nanostructures: Materials, Processes, and Applications to Electronics. Chem. Rev..

[10] Walther A., Müller A.H.E. (2013). Janus Particles: Synthesis, Self-Assembly, Physical Properties, and Applications. Chem. Rev..

[11] Park J., Jang S., Kim J.K. (2015). Morphology and Microphase Separation of Star Copolymers. J. Polym. Sci. B..

[12] Ren J.M., Mckenzie T.G., Fu Q., Wong E.H.H., Xu J., An J., Shanmugam S., Davis T.P., Boyer C., Qiao G.G. (2016). Star Polymers. Chem. Rev..

[13] Higashihara T. (2017). Precise Synthesis of Block and Miktoarm Star-Branched Polymers Containing Polythiophene Segments with Low Dispersity by Combination of Living Anionic Polymerization and Catalyst Transfer Polymerization Systems. Macromol. Chem. Phys..

[14] Bates C.M., Bates F.S. (2017). Block Polymers-Pure Potential. Macromolecules.

[15] Higashihara T., Sugiyama K., Yoo H.-S., Hayashi M., Hirao A. (2010). Combining Livimg Anionic Polymerization with Branching Reactions in an Iterative Fashion to Design Branched Polymers. Macromol. Rapid Commun..

[16] Hirao A., Murano K., Oie T., Uematsu M., Goseki R., Matsuo Y. (2011). Chain-End- and In-Chain-Functionalized AB Diblock Copolymers as Key Building Blocks in the Synthesis of Well-Defined Architectural Polymers. Polym. Chem..

[17] Hirao A., Hayashi M., Higashihara T., Hadjichristidis N., Hadjichristidis N., Hirao A., Tezuka Y., Du Prez F. (2011). Recent Developments in Miktoarm Star Polymers with More than Three Different Arms. Complex Macromolecular Architectures: Synthesis, Characterization, and Self-Assembly.

[18] Higashihara T., Hayashi M., Hirao A. (2011). Synthesis of Well-Defined Star-Branched Polymers by Stepwise Iterative Methodology Using Living Anionic Polymerization. Prog. Polym. Sci..

[19] Ito S., Goseki R., Ishizone T., Hirao A. (2013). Successive Synthesis of Well-Defined Multiarmed Miktoarm Star Polymers by Iterative Methodology Using Living Anionic Polymerization. Eur. Polym. J..

[20] Hirao A., Goseki R., Ishizone T. (2014). Advances in Living Anionic Polymerization: From Functional Monomers, Polymerization Systems, to Macromolecular Architectures. Macromolecules.

[21] Birshtein T.M., Polotsky A.A., Abetz V. (2004). Theory of the Lamellar Superstructure of an ABC 3-Miktoarm Star-Terpolymer. Macromol. Theory Simul..

[22] Takano A., Wada S., Sato S., Araki T., Hirahara K., Kazama T., Kawahara S., Isono Y., Ohno A., Tanaka N. (2004). Observation of Cylinder-Based Microphase-Separated Structures from ABC Star-Shaped Terpolymers Investigated by Electron Computerized Tomography. Macromolecules.

[23] Hayashida K., Takano A., Arai S., Shiohara Y., Amemiya Y., Matsushita Y. (2006). Systematic Transitions of Tiling Patterns Formed by ABC Star-Shaped Terpolymers. Macromolecules.

[24] Li Z., Hillmyer M.A., Lodge T.P. (2006). Morphologies of Multicompartment Micelles Formed by ABC Miktoarm Star Terpolymers. Langmuir.

[25] Li Z., Hillmyer M.A., Lodge T.P. (2006). Laterally Nanostructured Vesicles, Polygonal Bilayer Sheets, and Segmented Wormlike Micelles. Nano Lett..

[26] Walther A., Müller A.H.E. (2009). Formation of Hydrophobic Bridges between Multicompartment Micelles of Miktoarm Star Terpolymers in Water. Chem. Commun..

[27] Gitsas A., Floudas G., Mondeshki M., Lieberwirth I., Spiess H.W., Iatrou H., Hadjichristidis N., Hirao A. (2010). Hierarchical Self-Assembly and Dynamics of a Miktoarm Star *chimera* Composed of Poly(γ–benzyl–l–glutamate), Polystyrene, and Polyisoprene. Macromolecules.

[28] Junnila S., Houbenov N., Hanski S., Iatrou H., Hirao A., Hadjichristidis N., Ikkala O. (2010). Hierarchical Smectic Self-Assembly of an ABC Miktoarm Star Terpolymer with a Helical Polypeptide Arm. Macromolecules.

[29] Goseki R., Hirao A., Kakimoto M., Hayakawa T. (2013). Cylindrical Nanostructure of Rigid-Rod POSS-Containing Polymethacrylate from a Star-Branched Block Copolymer. ACS Macro Lett..

[30] Iatrou H., Hadjichristidis N. (1992). Synthesis of a Model 3-Miktoarm Star Terpolymer. Macromolecules.

[31] Fujimoto T., Zang H., Kazama T., Isono Y., Hasegawa H., Hashimoto T. (1992). Preparation and Characterization of Novel Star-shaped Copolymers Having Three Different Branches. Polymer.

[32] Iatrou H., Hadjichristidis N. (1993). Synthesis and Characterization of Model 4-Miktoarm Star Co- and Quarterpolymers. Macromolecules.

[33] Hückstädt H., Abetz V., Stadler R. (1996). Synthesis of a Polystyrene-*arm*-Polybutadiene-*arm*-Poly(methyl methacrylate) Triarm Star Copolymer. Macromol. Rapid Commun..

[34] Hadjichristidis N. (1999). Synthesis of Miktoarm Star (μ-Star) Polymers. J. Polym. Sci. A.

[35] Hirao A., Tokuda Y. (2003). Synthesis of Well-Defined Star-Branched Polymers by Coupling Reactions of Polymer Anions Consisting of Two Polymer Chains with Chain-End-Multifunctionalized Polystyrenes with Benzyl Bromide Moieties. Macromolecules.

[36] Hirao A., Kawasaki K., Higashihara T. (2004). Synthesis of Well-Defined Star-Branched Polymers by Coupling Reaction of Star-Branched Polymer Anions Comprised of Three Polymer Segments with Chain-End-Functionalized Polystyrenes with a Definite Number of Benzyl Bromide Moieties. Macromolecules.

[37] Higashihara T., Hirao A. (2004). Synthesis of Asymmetric Star-Branched Polymers Consisting of Three or Four Different Segments in Composition by Means of Living Anionic Polymerization with a New Dual-Functionalized 1,1-Bis(3-chloromethylphenyl)ethylene. J. Polym. Sci. A.

[38] Mavroudis A., Hadjichristidis H. (2006). Synthesis of Well-Defined 4-Miktoarm Star Quarterpolymers (4 μ-SIDV) with Four Incompotible Arms: Polystyrene (S), Polyisoprene-1,4 (I), Poly(dimethylsiloxane) (D), and Poly(2-vinylpyridine) (V). Macromolecules.

[39] Wang X., Xia J., He J., Yu F., Li A., Xu J., Lu H., Yang Y. (2006). Synthesis and Characterization of ABC-type Star and Linear Block Copolymers of Styrene, Isoprene, and 1,3-Cyclohexadiene. Macromolecules.

[40] Fragouli P., Iatrou H., Hadjichristidis N., Sakurai T., Matsunaga Y., Hirao A. (2006). Synthesis of Well-Defined Miktoarm Star Polymers of Poly(dimethylsiloxane) by the Combination of Chlorosilane and Benzyl Chloride Linking Chemistry. J. Polym. Sci. A.

[41] Wang X., He J., Yang Y. (2007). Synthesis of ABCD-Type Miktoarm Star Copolymers and Transformation into Zwitterionic Star Copolymers. J. Polym. Sci. A.

[42] Karatzas A., Iatrou H., Hadjichristidis N., Inoue K., Sugiyama K., Hirao A. (2008). Complex Macromolecular *Chimeras*. BioMacromolecules.

[43] Hanisch A., Schmalz H., Müller A.H.E. (2012). A Modular Route for the Synthesis of ABC Miktoarm Star Terpolymers via a New Alkyne-Substituted Diphenylethylene Derivative. Macromolecules.

[44] Rho Y., Kim C., Higashihara T., Jin S., Jung J., Shim T.J., Hirao A., Ree M. (2013). Complex Self-Assembled Morphologies of Thin Films of an Asymmetric A_3_B_3_C_3_ Star Polymer. ACS Macro Lett..

[45] Aissou K., Choi H.K., Nunns A., Manners I., Ross C.A. (2013). Ordered Nanoscale Archimedean Tilings of a Templated 3-Miktoarm Star Terpolymer. Nano Lett..

[46] Ye C., Zhao G., Zhang M., Du J., Zhao Y. (2012). Precise Synthesis of ABCDE Star Quintopolymers by Combination of Controlled Polymerization and Azide-Alkyne Cycloaddition Reaction. Macromolecules.

[47] Higashihara T., Nagura M., Inoue K., Haraguchi N., Hirao A. (2005). Successive Synthesis of Well-Defined Star-Branched Polymers by a New Iterative Approach Involving Coupling and Transformation Reactions. Macromolecules.

[48] Higashihara T., Inoue K., Nagura M., Hirao A. (2006). Successive Synthesis of Well-Defined Star-Branched Polymers by a New Iterative Approach Based on Living Anionic Polymerization. Macromol. Res..

[49] Ito S., Goseki R., Senda S., Hirao A. (2012). Precise Synthesis of Miktoarm Star Polymers by Using a New Dual-Functionalized 1,1-Diphenylethylene Derivative in Conjunction with Living Anionic Polymerization System. Macromolecules.

[50] Goseki R., Ozama Y., Akemine E., Ito S., Ehara S., Hirao A. (2013). Precise Synthesis of Poly(methacrylate)-Based Miktoarm Star Polymers by a New Stepwise Iterative Methodology Using a Formyl-Functionalized 1,1-Diphenylethylene Derivative. Polymer.

[51] Ito S., Goseki R., Manners I., Ishizone T., Hirao A. (2015). Successive Synthesis of Multicomponent Star-Branched Polymers by New Iterative Methodology Based on Linking Reaction between Block Copolymer In-Chain Anion and α-Phenylacrylate-Functionalized Polymer. Macromol. Chem. Phys..

[52] Goseki R., Ito S., Akemine E., Hirao A. (2016). Facile Synthesis of Multiarmed and Multicomponent Star Polymers by a New Iterative Methodology Using (Formyl-Protected 1,3-Dioxolane)-End-Functionalized Polymer Anions. Polym. Chem..

[53] Ito S., Goseki R., Ishizone T., Senda S., Hirao A. (2013). Successive Synthesis of Miktoarm Star Polymers Having up to Seven Arms by a New Iterative Methodology Based on Living Anionic Polymerization Using a Trifunctional Lithium Reagent. Macromolecules.

[54] Six J.L., Gnanou Y. (1995). From Star-Shaped to Dendritic Poly(ethylene oxide)s: Toward Increasingly Branched Architectures by Anionic Polymerization. Macromol. Symp..

[55] Hirao A., Sugiyama K., Tsunoda Y., Matsuo A. (2006). Precise Synthesis of Well-Defined Dendrimer-Like Star-Branched Polymers by Iterative Methodology Based on Living Anionic Polymerization. J. Polym. Sci. A.

[56] Taton D., Feng X., Gnanou Y. (2007). Dendrimer-Like Polymers: A New Class of Structurally Precise Dendrimers with Macromolecular Generations. New J. Chem..

[57] Voit B.I., Lederer A. (2009). Hyperbranched and Highly Branched Polymer Architectures–Synthetic Strategies and Major Characterization Aspects. Chem. Rev..

[58] Konkolewicz D., Monteiro M.J., Perrier S. (2011). Dendritic and Hyperbranched Polymers from Macromolecular Units: Elegant Approaches to the Synthesis of Functional Polymers. Macromolecules.

[59] Yoo H.S., Hirao A., Hadjichristidis N., Hirao A., Tezuka Y., Du Prez F. (2011). Precise Synthesis of Dendrimer-Like Star-Branched Polymers, A New Class of Well-Defined Hyperbranched Polymers. Complex Macromolecular Architectures: Synthesis, Characterization, and Self-Assembly.

[60] Trollsås M., Hedrick J.L. (1998). Dendrimer-like Star Polymers. J. Am. Chem. Soc..

[61] Hedrick J.L., Trollsås M., Hawker C.J., Athoff B., Claesson H., Heise A., Miller R.D., Mecerreyes D., Jérôme R., Dubois P. (1998). Dendrimer-like Star Block and Amphiphilic Copolymers by Combination of Ring Opening and Atom Transfer Radical Polymerization. Macromolecules.

[62] Trollsås M., Atthof B., Würsch A., Hedrick J.L., Pople J.A., Gast A.P. (2000). Constitutional Isomers of Dendrimer-like Star Polymers: Design, Synthesis, and Conformational and Structural Properties. Macromolecules.

[63] Hedrick J.L., Magbitang T., Connor E.F., Glauser T., Volksen W., Hawker C.J., Lee V.Y., Miller R.D. (2002). Application of Complex Macromolecular Architectures for Advanced Microelectronic Materials. Eur. J..

[64] Chalari I., Hadjichristidis N. (2002). Synthesis of well-defined secondd-generation dendritic polymers of isoprene (I) and styene (S): (S_2_I)_3_, (SI’I)_3_, (I”I’I)_3_, and (I’_2_I)_4_. J. Polym. Sci. A.

[65] Percec V., Barboiu B., Grigoras C., Bera T.K. (2003). Universal Iterative Strategy for the Divergent Synthesis of Dendritic Macromolecules from Irreversible TERminator Multifunctional INItiator (TERMINI). J. Am. Chem. Soc..

[66] Percec V., Grigonas C., Bera T.K., Barboiu B., Bissel P. (2005). Accelerated iterative strategy for the divergent synthesis of dendritic macromolecules using a combination of living radical polymerization and an irreversible terminator multifunctional initiator. J. Polym. Sci. A.

[67] Hutchings L.R., Roberts-Bleming S.J. (2006). DendriMacs. Well-Defined Dendritically Branched Polymers Synthesized by an Iterative Convergent Strategy Involving the Coupling Reaction of AB_2_ Macromonomers. Macromolecules.

[68] Orfanou K., Iatrou H., Lohse D.J., Hadjichristidis N. (2006). Synthesis of Well-Defined Second (G-2) and Third (G-3) Generation Dendritic Polybutadienes. Macromolecules.

[69] Hutchings L.R. (2008). DendriMacs and HyperMacs—Emerging as More than Just Model Branched Polymers. Soft Matter.

[70] Urbani C.N., Bell C.A., Lonsdale D., Whittaker M.R., Monteiro M.J. (2008). Self-Assembly of Amphiphilic Polymeric Dendrimers Synthesized with Selective Degradable Linkages. Macromolecules.

[71] Urbani C.N., Bell C.A., Whittaker M.R., Monteiro M.J. (2008). Convergent Synthesis of Second Generation AB-Type Miktoarm Dendrimers Using “Click” Chemistry Catalyzed by Copper Wire. Macromolecules.

[72] Deffieux A., Schappacher M., Hirao A., Watanabe T. (2008). Synthesis and AFM Strucrtural Imaging of Dendrimer-Like Star-Branched Polystyrenes. J. Am. Chem. Soc..

[73] Feng X., Taton D., Chaikof E.L., Gnanou Y. (2009). Fast Access to Dendrimer-like Poly(ethylene oxide)s through Anionic Ring-Opening Polymerization of Ethylene Oxide and Use of Nonprotected Glycidol as Branching Agent. Macromolecules.

[74] Ruymbeke E.V., Muliawan E.B., Hatzikiriakos S.G., Watanabe T., Hirao A., Vlassopoulos D. (2010). Viscoelasticity and Extensional Rheology of Model Cayley-Tree Polymers of Different Generations. J. Rheol..

[75] Hong L., Yang S., He J. (2015). Molecular Engineering of Branched Polymers through 1,1-Diphenylethylene Chemistry and Anionic Polymerization. Eur. Polym. J..

[76] Lu D., Hossain M.D., Jia Z., Monteiro M.J. (2015). One-Pot Orthogonal Copper-Catalyzed Synthesis and Self-Assembly of l-Lysine-Decorated Polymeric Dendrimers. Macromolecules.

[77] Makelane H.R., Tovide O., Sunday C.E., Waryo T., Iwuoha E.I. (2015). Electrochemical Interrogation of G3-Poly(propylenethiophenoimine) Dendritic Star Polymer in Phenanthrene Sensing. Sensors.

[78] Tungala K., Adhikary P., Azmeera V., Kumar K., Ramesh K., Krishnamoorthi S. (2016). Dendrimer-like Star Polymer Based on β-Cyclodextrin with ABC Type Miktoarms. RSC Adv..

[79] Zhu S., Xia R., Chen P., Yang B., Miao J., Zheng Z., Su L., Qian J., Cao M., Feng X. (2017). High-Efficiency Synthesis of Dendrimer-Like Poly(ethylene oxide) via “Arm-First” Approach. J. Polym. Res..

[80] Feng X., Taton D., Chaikof E.L., Gnanou Y. (2005). Toward an Easy Access to Dendrimer-like Poly(ethylene oxide)s. J. Am. Chem. Soc..

[81] Matmour R., Gnanou Y. (2008). Combination of an Anionic Terminator Multifunctional Initiator and Divergent Carbanionic Polymerization: Application to the Synthesis of Dendrimer-Like Polymers and of Asymmetric and Miktoarm Stars. J. Am. Chem. Soc..

[82] Matsuo A., Watanabe T., Hirao A. (2004). Synthesis of Well-Defined Dendrimer-like Branched Polymers and Block Copolymer by the Iterative Approach Involving Coupling Reaction of Living Anionic Polymer and Functionalization. Macromolecules.

[83] Hirao A., Matsuo A., Watanabe T. (2005). Precise Synthesis of Dendrimer-like Star-Branched Poly(methyl methacrylate)s up to Seventh Generation by an Iterative Divergent Approach Involving Coupling and Transformation Reactions. Macromolecules.

[84] Watanabe T., Tsunoda Y., Matsuo A., Hirao A. (2006). Synthesis of Dendrimer-Like Star-branched Poly(methyl methacrylate)s of generation consisting of Four Branched Polymer Chains at Each Junction by Iterative Methodology Involving Coupling and Transformation reactions. Macromol. Symp..

[85] Hirao A., Watanabe T., Ishizu K., Ree M., Jin K.S., Deffieux A., Schappacher M., Carlotti S. (2009). Precise Synthesis and Characterization of Fourth-Generation Dendrimer-like Star-Branched Poly(methyl methacrylate)s and Block Copolymers by Iterative Methodology Based on Living Anionic Polymerization. Macromolecules.

[86] Yoo H.S., Watanabe T., Hirao A. (2009). Precise Synthesis of Dendrimer-Like Star-Branched Polystyrenes and Block Copolymers Composed of Polystyrene and Poly(methyl methacrylate) Segments by an Iterative Methodology Using Living Anionic Polymerization. Macromolecules.

[87] Yoo H.S., Watanabe T., Matsunaga Y., Hirao A. (2012). Precise Synthesis of Dendrimer-like Star-Branched Poly(*tert*-butyl methacrylate)s and Their Block Copolymers by a Methodology Combining α-terminal-Functionalized Living Anionic Polymers with a Specially Designed Linking Reaction in an Iterative Fashion. Macromolecules.

[88] Paraskeva S., Hadjichristidis N. (2000). Synthesis of an Exact Graft Copolymer of Isoprene and Styrene with Two Branches. J. Polym. Sci. A.

[89] Quirk R.P., Hsieh H.L., Quirk R.P. (1996). Graft Copolymers. Anionic Polymerization: Principles and Practical Applications.

[90] Uhrig D., Mays J.W. (2011). Synthesis of Well-Defined Multigraft Copolymers. Polym. Chem..

[91] Ito S., Goseki R., Ishizone T., Hirao A. (2014). Synthesis of Well-Controlled Graft Polymers by Living Anionic Polymerization towards Exact Graft Polymers. Polym. Chem..

[92] Hirao A., Watanabe T., Kurokawa R. (2009). Precise Synthesis of Exact Graft Polystyrenes with Branches from Two to Five in Number by Iterative Methodology Based on Living Anionic Polymerization. Macromolecules.

[93] Hirao A., Murano K., Kurokawa R., Watanabe T., Sugiyama K. (2009). Precise Synthesis of Exact Graft Copolymers, Poly(methyl methacrylate)-exact graft-polystyrene, by Iterative Methodology Using a Specially Designed In-Chain-Functionalized AB Diblock Copolymer Anion. Macromolecules.

[94] Hirao A., Murano K., Abouelmagd A., Uematsu M., Ito S., Goseki R., Ishizone T. (2011). General and Facile Approach to Exact Graft Copolymers by Iterative Methodology Using Living Anionic In-Chain-Functionalized AB Diblock Copolymers as Key Building Blocks. Macromolecules.

[95] Hirao A., Uematsu M., Kurokawa R., Ishizone T., Sugiyama K. (2011). Facile Synthetic Approach to Exact Graft (Co)polymers and Double-Tailed Polystyrene: Linking Reaction of Living Anionic Polymers with Specially Designed In-Chain-Multifunctionalized Polystyrenes. Macromolecules.

[96] Ito S., Ishizone T., Hirao A. (2015). Precise Synthesis of New Exactly Defined Graft Copolymers Made up of Poly(alkyl methacrylate)s by Iterative Methodology Using Living Anionic Polymerization. Macromolecules.

[97] Ito S., Senda S., Ishizone T., Hirao A. (2016). Synthesis of Exactly-Defined Multigraft Polymers with Two or More Graft Chains per Branch Point by a New Iterative Methodology. Polym. Chem..

[98] Hsieh H.L., Quirk R.P., Hsieh H.L., Quirk R.P. (1996). Block Copolymers. Anionic Polymerization: Principles and Practical Applications.

[99] Sugiyama K., Oie T., El-Magd A.A., Hirao A. (2010). Synthesis of Well-Defined (AB)*_n_* Multiblock Copolymers Composed of Polystyrene and Poly(methyl methacrylate) Segments Using Specially Designed Living AB Diblock Copolymer Anion. Macromolecules.

[100] Hirao A., Matsuo Y., Oie T., Goseki R., Ishizone T., Sugiyama K., Göschel A.H., Müller A.H.E. (2011). Facile Synthesis of Triblock Co- and Terpolymers of Styrene, 2-Vinylpyridine, and Methyl Methacrylate by a New Methodology Combining Living Anionic Diblock Copolymers with a Specially Designed Linking Reaction. Macromolecules.

[101] Matsuo Y., Konno R., Ishizone T., Goseki R., Hirao A. (2013). Precise Synthesis of Block Polymers Composed of Three or More Blocks by Specially Designed Linking Methodologies in Conjunction with Living Anionic Polymerization System. Polymers.

[102] Matsuo Y., Oie T., Goseki R., Ishizone T., Sugiyama K., Hirao A. (2013). Precise Synthesis of New Triblock Co- and Terpolymers by a Methodology Combining Living Anionic Polymers with a Specially Designed Linking Reaction. Macromol. Symp..

[103] Matsuo Y., Konno R., Goseki R., Ishizone T., Hirao A. (2016). Tailored Synthesis of Triblock Co- and Terpolymers Composed of Synthetically Difficult Sequence Orders by Combining Living Anionic Polymerization with Specially Designed Linking Reaction. Macromol. Chem. Phys..

[104] Gerogios T., Pitsikalis M., Hadjichristidis N., Hirao A. (2015). Block Copolymers by Anionic Polymerization: Recent Synthetic Routes and Developments. Anionic Polymerization; Principles, Practice, Strength, Consequences and Application.

[105] Stadler R., Auschra C., Beckmann J., Kraqppe U., Voight-Martin I., Leibler L. (1995). Morphology and Thermodynamics of Symmetric Poly(A–*block*–B–*block*–C) Triblock Copolymers. Macromolecules.

[106] Giebeler E., Stadler R. (1997). ABC Triblock Polyampholytes Containing a Neutral Hydrophobic Block, a Polyacid and a Polybase. Macromol. Chem. Phys..

[107] Ludwings S., Böker A., Abetz V., Müller A.H.E., Krausch G. (2003). Phase Behavior of Linear Polystyrene–*block*–Poly(2-vinylpyridine)–*block*–Poly(*tert*-butyl methacrylate) Triblock Terpolymers. Polymer.

[108] Schacher F., Walther A., Ruppel M., Drechsler M., Müller A.H.E. (2009). Multicompartment Core Micelles of Triblock Terpolymers in Organic Media. Macromolecules.

[109] Schacher F., Yuan J., Schoberth H.G., Müller A.H.E. (2010). Synthesis, Characterization, and Bulk Crosslinking of Polybutadiene–*block*–Poly(2-vinylpyridine)–*block*–Poly(*tert*-butyl methacrylate) Block Terpolymers. Polymer.

[110] Watanabe H., Shimura T., Kotaka T., Tirrell M. (1993). Synthesis, Characterization, and Surface Structures of Styrene-2-Vinylpyridine-Butadiene Three-Block Polymers. Macromolecules.

[111] Bellas V., Iatrou H., Hadjichristidis N. (2000). Controlled Aninonic Polymerization of Hexamethylcyclotrisiloxane. Model Linear and Miktoarm Star Co- and Terpolymers of Dimethylsiloxane with Styrene and Isoprene. Macromolecules.

[112] Takahashi K., Hasegawa H., Hashimoto T., Bellas V., Iatrou H., Hadjichristidis N. (2002). Four-Phase Triple Coaxial Cylindrical Microdomain Morphology in a Linear Tetrablock Quaterpolymer of Styrene, Isoprene, Dimethylsiloxane, and 2-Vinylprydine. Macromolecules.

[113] Takano A., Soga K., Asari T., Suzuki J., Arai S., Saka H., Matsushita Y. (2003). Observation of Four-Phase Lamellar Structure from a Tetrablock Quarterpolymer of the ABCD Type. Macromolecules.

[114] Liu F., Eisenberg A. (2003). Synthesis of Poly(*tert*-butyl acrylate)–*block*–Polystyrene–*block*–Poly(4-vinylpyridine) by Living Anionic Polymerization. Angew. Chem. Int. Ed..

[115] Fragouli P.G., Iatrou H., Hadjichristidis N. (2004). Synthesis and Characterization of Linear Tetrablock Quaterpolymers of Styrene, Isoprene, Dimethylsiloxane, and 2-Vinylpyridine. J. Polym. Sci. A.

[116] Bellas V., Rehahn M. (2007). Universal Methodology for Block Copolymer Synthesis. Macromol. Rapid Commun..

[117] Fragouli P., Iatrou H., Lohse D.J., Hadjichristidis N. (2008). Linear Pentablock Quintopolymers (I-SIDMV) with Five Incompatible Blocks: Polystyrene, Polyisoprene-1,4, Poly(dimethylsiloxane), Poly(*tert*-butyl methacrylate), and Poly(2-vinylpyridine). J. Polym. Sci. A.

[118] Bellas V., Rehahn M. (2009). Block Copolymer Synthesis via Chemoselective Stepwise Coupling Reactions. Macromol. Chem. Phys..

[119] Touris A., Lee S., Hillmyer M.A., Bates F.S. (2012). Synthesis of Tri- and Multiblock Polymers with Asymmetric Poly(ethylene Oxide) End Blocks. ACS Macro Lett..

[120] Touris A., Chanpuriya S., Hillmyer M.A., Bates F.S. (2014). Synthetic Strategies for the Generation of ABCA’ Type Asymmetric Tetrablock Terpolymers. Polym. Chem..

[121] Lin W., Fu Q., Zhang Y., Huang J. (2008). One-Pot Synthesis of ABC Type Triblock Copolymers via a Combination of “Click Chemistry” and Atom Transfer Nitroxide Radical Coupling Chemistry. Macromolecules.

[122] Reinicke S., Schmalz H. (2011). Combination of Living Anionic Polymerization and ATRP via “Click” Chemistry as a Versatile Route to Multiple Responsive Triblock Terpolymers and Corresponding Hydrogels. Colloid Polym. Sci..

[123] Bernaerts K.V., Du Prez F.E. (2006). Dual/Heterofunctional Initiators for the Combination of Mechanistically Distinct Polymerization Techiques. Prog. Polym. Sci..

[124] Yagci Y., Tasdelen M.A. (2006). Mechanistic Transformations Involving Living and Controlled/Living Polymerization Methods. Prog. Polym. Sci..

[125] Inglis A.J., Barner-Kowollik C., Hadjichristidis N., Hirao A., Tezuka Y., Du Prez F. (2011). Ultrarapid Approaches to Mild Macromolecular Conjugation. Complex Macromolecular Architectures: Synthesis, Characterization, and Self-Assembly.

[126] Matyjaszewski K., Tsarevsky N.V. (2014). Macromolecular Engineering by Atom Transfer Radical Polymerization. J. Am. Chem. Soc..

